# Supplementary material for: Solvent concentration at 50% protein unfolding may reform enzyme stability ranking and process window identification
Source: Nat Commun. 2024 Jun 26;15:5420. doi: 10.1038/s41467-024-49774-0 (PMC11208486; doi:10.1038/s41467-024-49774-0)
Supplement: Supplementary file 1 — Supplementary Information [file 41467_2024_49774_MOESM1_ESM.pdf]

Supplementary Information

Solvent concentration at 50% protein unfolding may reform enzyme stability ranking and process window identification

Frieda A. Sorgenfrei[1], Jeremy J. Sloan[2], Florian Weissensteiner[1,3], Marco Zechner[1], Niklas A. Mehner[2], Thomas L. Ellinghaus[2], Doreen Schachtschabel[2], Stefan Seemayer[2,*], Wolfgang Kroutil[1,3,4,5,*]

Correspondence: stefan.seemayer@basf.com, wolfgang.kroutil@uni-graz.at

Supplementary Table 1 List of sequence identifier, origin and molecular weight of ene reductases and transminases.

pEG[a]	Enzyme	Accesion No/Uniprot ID	Origin	MW [kDa]
516	NCR	Q5NLA1	*Zymomonas mobilis* subsp. mobilis	39.5
722	PpXenB	Q88PD0	*Pseudomonas putida*	37.8
146	XenB	Q9RPM1	*Pseudomonas fluorescens*	37.6
147	NerA	O31246	*Rhizobium radiobacter*	39.7
360	OYE1	Q02899	*Saccharomyces pastorianus*	44.9
723	ChrOYE1	A0A0U2H4S5	*Chryseobacterium* sp. CA49	39.4
724	RmER	Q1LDQ5	*Cupriavidus metallidurans*	40.1
725	DrER	Q9RSD4	*Deinococcus radiodurans*	39.6
368	YqjM	P54550	*Bacillus subtilis*	37.5
145	XenA	Q9R9V9	*Pseudomonas putida*	39.8
726	CrS/TsOYE	B0JDW3	*Thermus scotoductus*	38.0
727	YqiG	P54524	*Bacillus subtilis*	40.8
728	LacER	S4ZS69	*Lactobacillus paracasei*	42.4
29	ArS	PDB: 5G2P_A	*Arthrobacter citreus*	53.0
234	ArR	3WWH_A	*Arthrobacter sp.*	35.9

[a] pEG is the group internal plasmid number standing for plasmids of the elk group.

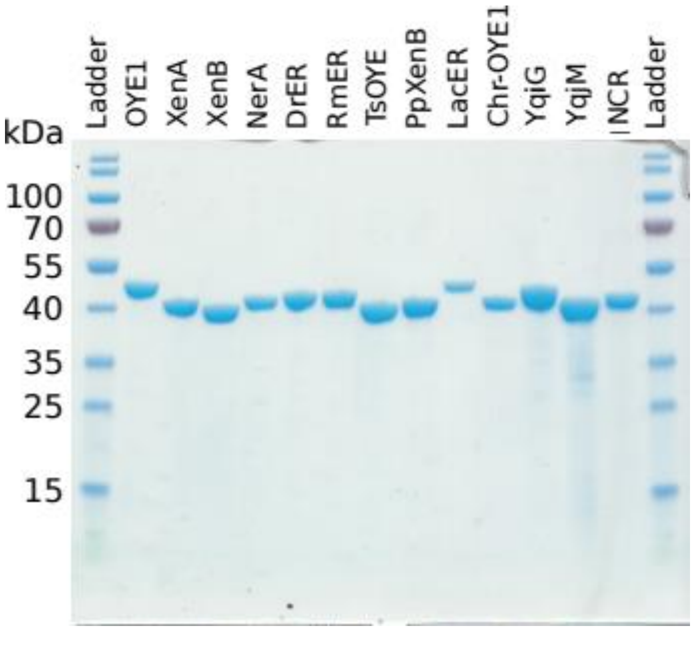

Supplementary Fig. 1 SDS gel of purified ene reductases.

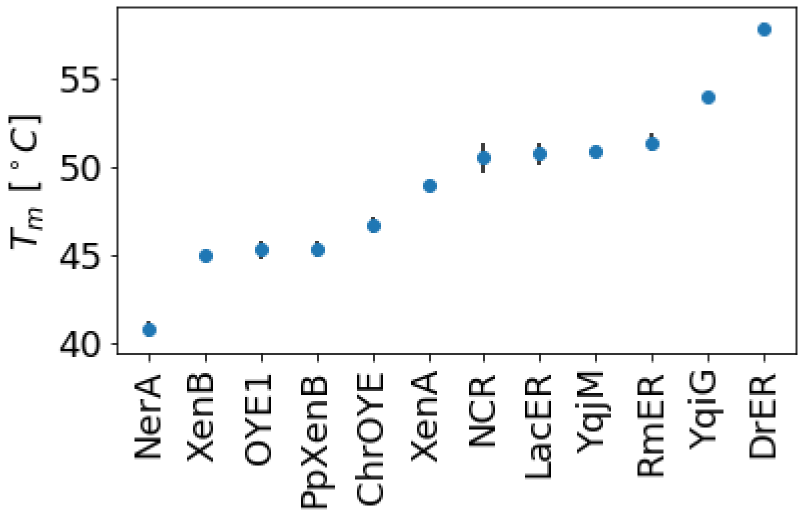

Supplementary Fig. 2 Melting temperature *T_m* of ene reductases in the absence of co-solvent. 1 mg mL^{-1} EREDs were measured in 50 mM sodium phosphate buffer pH 7.4 using ThermoFMN assay. Melting of TsOYE was larger than 90 °C which was above the measured range. Mean and standard deviation of the mean of 15 individual measurements (reference values of each co-solvent series) are shown here.

Supplementary Table 2 Melting temperature of ene reductases in the absence of co-solvent.

Enzyme	T_m	T_m literature
	°C	°C
ChrOYE1	46.7 ± 0.5	
DrER	57.9 ± 0.3	
LacER	51.0 ± 0.8	
NCR	50.5 ± 0.8	59.8[1]
NerA	40.7 ± 0.5	
OYE1	45.3 ± 0.5	
PpXenB	45.3 ± 0.5	
RmER	51.4 ± 0.5	
TsOYE	>90	
XenA	49.0 ± 0.0	50.4 [2]
XenB	45.0 ± 0.0	
YqiG	54.0 ± 0.0	
YqjM	50.9 ± 0.3	50 [3]

Note: 1 mg mL^{-1} enzyme in 50 mM sodium phosphate buffer, mean and standard deviation of the mean of 15 individual measurements (reference values of each co-solvent series).

For clarity the following Figures show the data of all EREDs, including the four presented in the main paper.

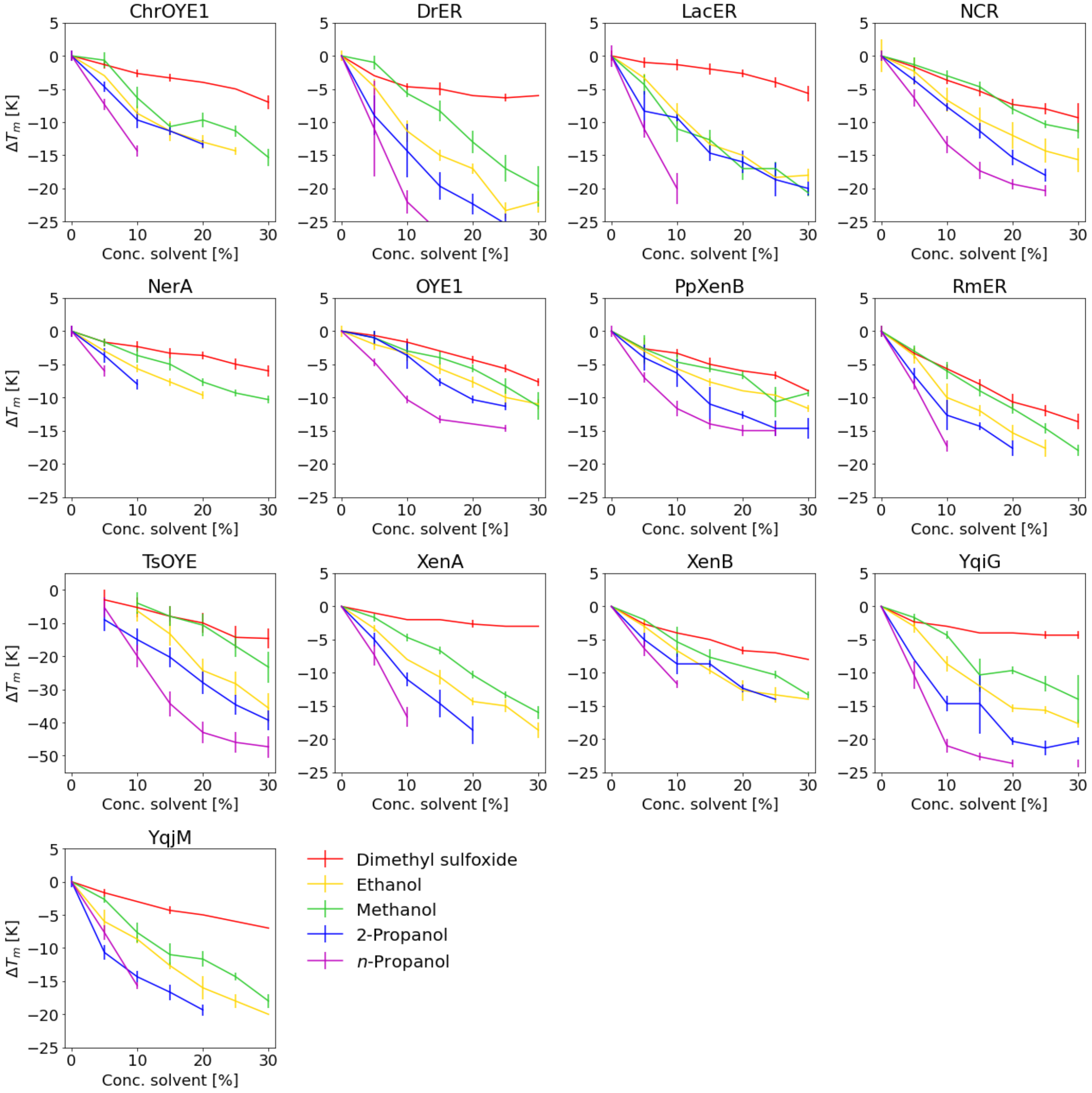

Supplementary Fig. 3 Change of melting temperature of ene reductases in the presence of certain amounts of co-solvents. The melting temperature was measured in 50 mM sodium phosphate buffer pH 7.4 using the ThermoFMN assay and the mean change of melting temperature of three replicates is given. The error bar marks the standard error of the mean using Gaussian error propagation. Note: All raw data that was used to obtain the derived values ΔT_m **Supplementary Table 5**.

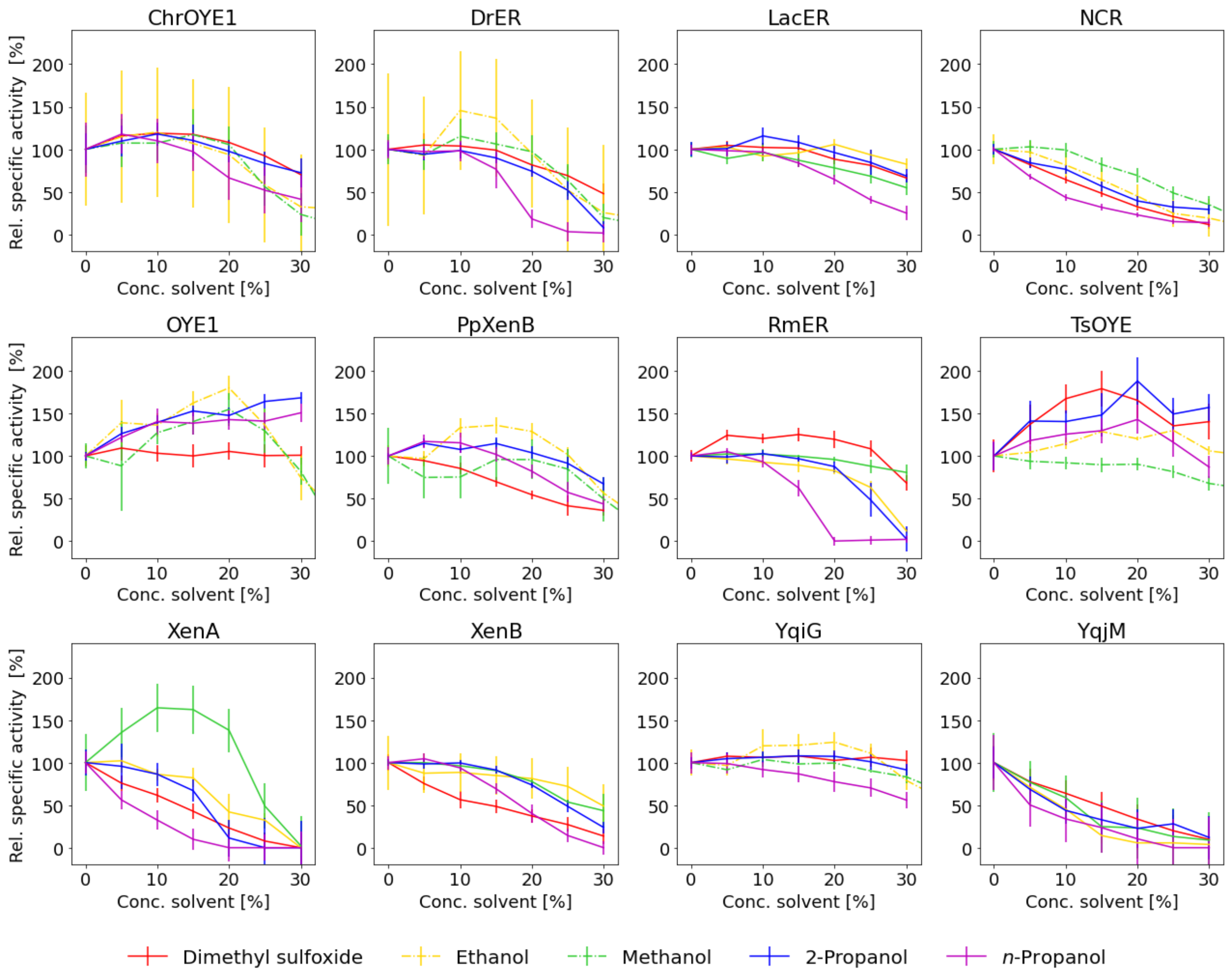

Supplementary Fig. 4 Relative specific activity of ene reductases in the presence of varied concentration of co-solvent. The initial activity was recorded under the same conditions as the melting temperature with the addition of substrate and cofactor (50 mM sodium phosphate buffer pH 7.4, 10 mM cyclohex-2-enone, 0.2 mM NAD(P)H). The values given here are normalized by setting the activity measured without any co-solvent (native conditions) to one. Given is the mean of three replicates. Values above one thus indicate an increased activity while values below one indicate a decreased activity relative to the activity at native conditions. The error bar marks the standard error of the mean using Gaussian error propagation. Note: All raw data that was used to obtain the derived relative specific activity can be found in **Supplementary Table 6**.

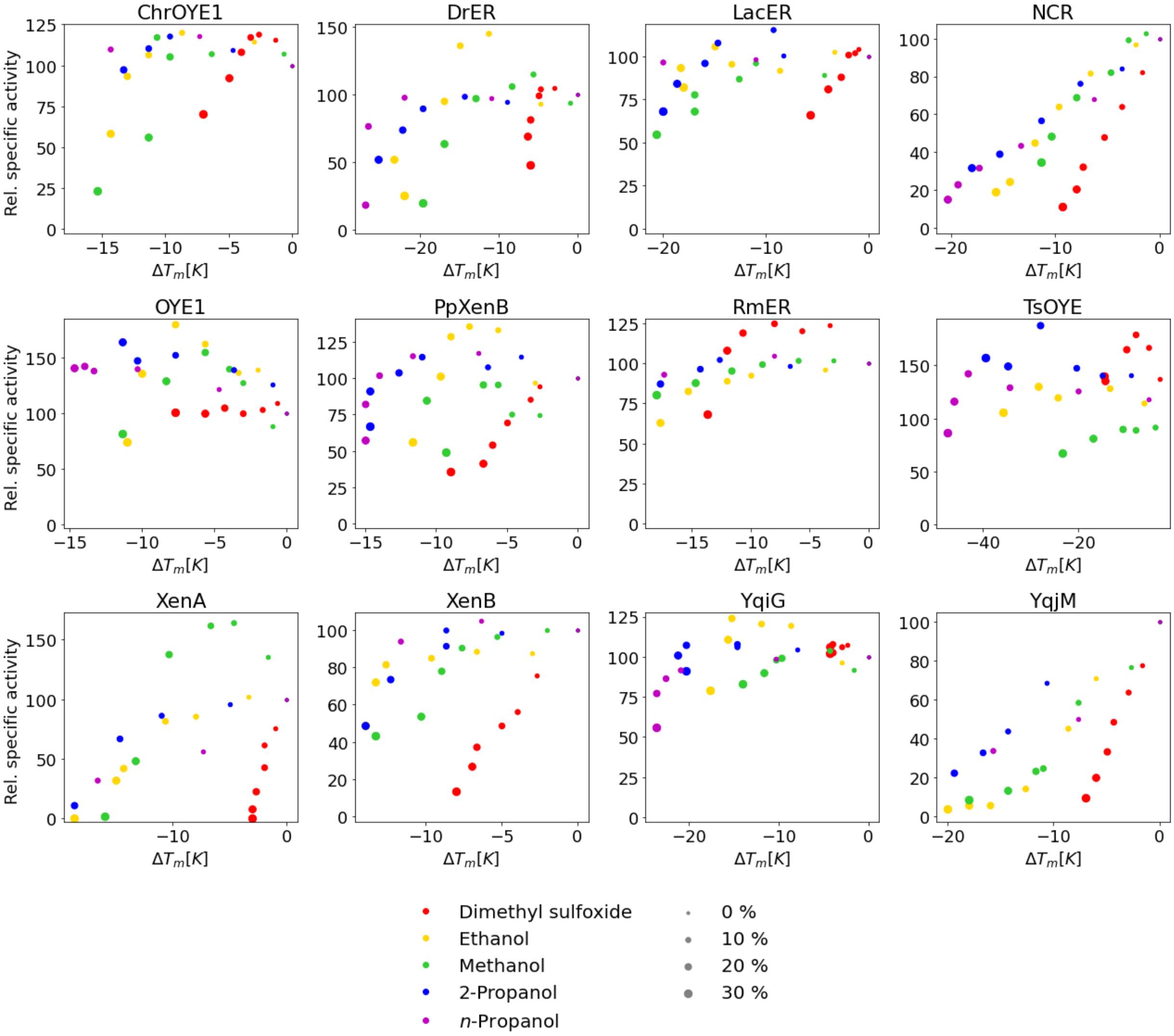

Supplementary Fig. 5 Comparison of relative specific activity and the change of melting temperature under the same conditions. These plots show a direct comparison of the change of melting temperature on the x-axis and the relative specific activity on the y-axis. The color of the dots represents which type of solvent was present and the size of the dots reflects on the concentration of solvent used. Please not that the measurement without solvent was measured as part of each solvent series. Note: All raw data that was used to obtain the derived values ΔT_m and relative specific activity can be found in **Supplementary Table 5** and **6**.

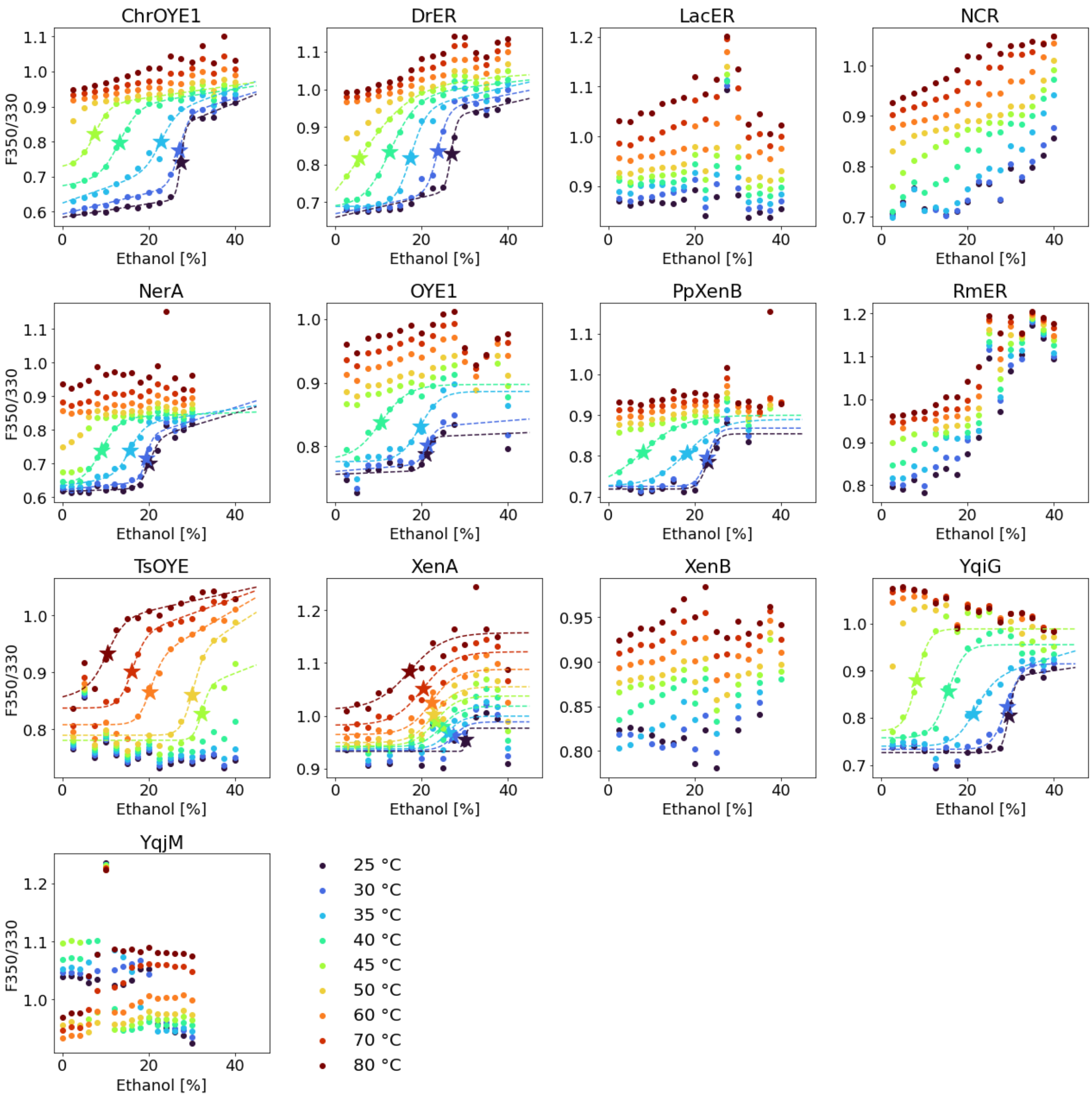

Supplementary Fig. 6 Unfolding curves induced by the presence of increasing amounts of ethanol. Unfolding of ene reductases in 50 mM sodium phosphate buffer pH 7.4 supplemented with increasing amounts of ethanol at different temperatures measured by nanoDSF (given as the ratio of the fluorescence at 350 and 330 nm [F350/330]). The dashed lines show fits of the recorded data to the two-state model of unfolding (**Equation (2)**). The star marks the unfolding concentration $c_{U_{50}}$.

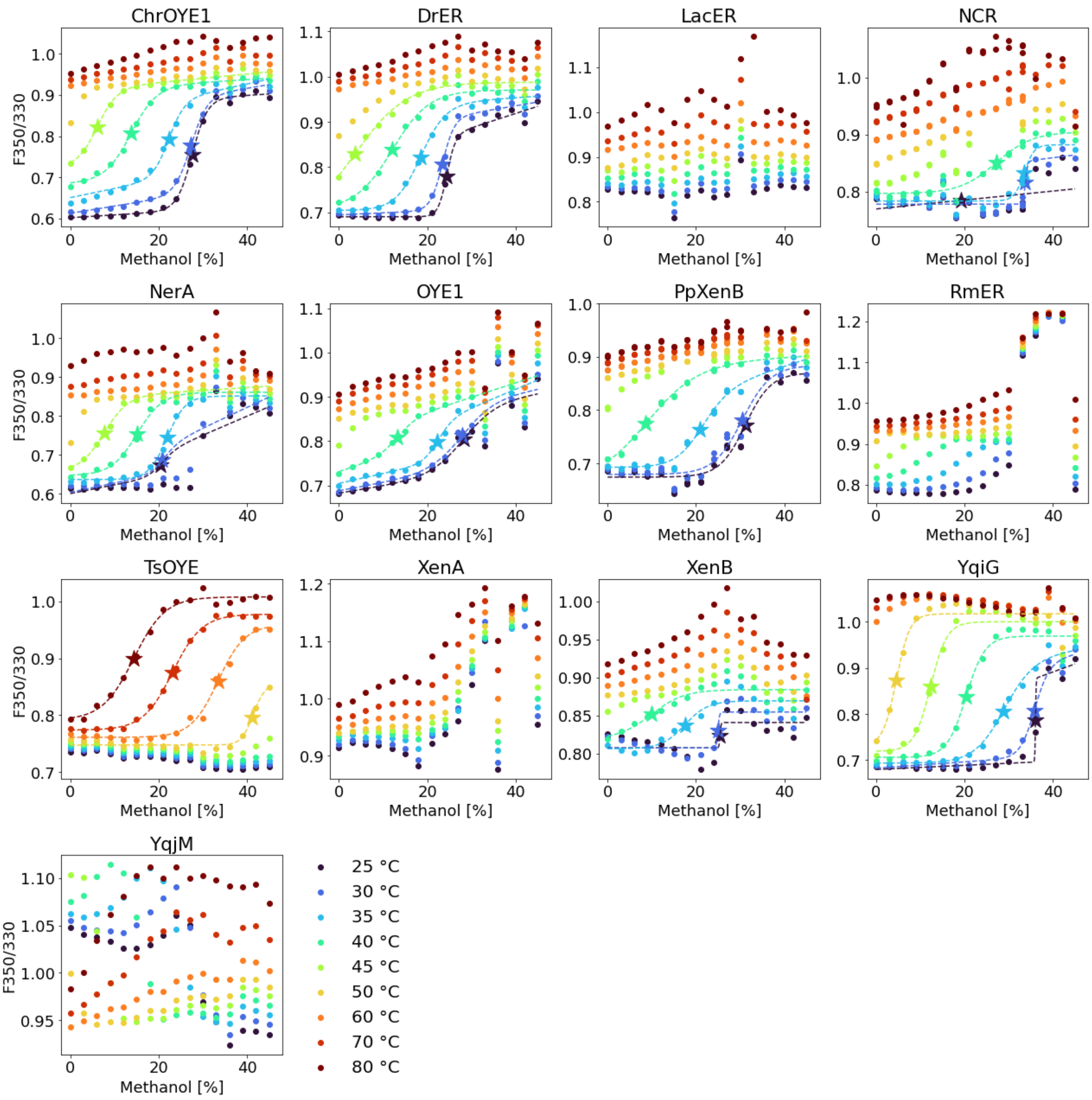

Supplementary Fig. 7 Unfolding curves induced by the presence of increasing amounts of methanol. Unfolding of ene reductases in 50 mM sodium phosphate buffer pH 7.4 supplemented with increasing amounts of methanol at different temperatures measured by nanoDSF (given as the ratio of the fluorescence at 350 and 330 nm [F350/330]). The dashed lines show fits of the recorded data to the two-state model of unfolding (**Equation (2)**). The star marks the unfolding concentration $c_{U_{50}}$.

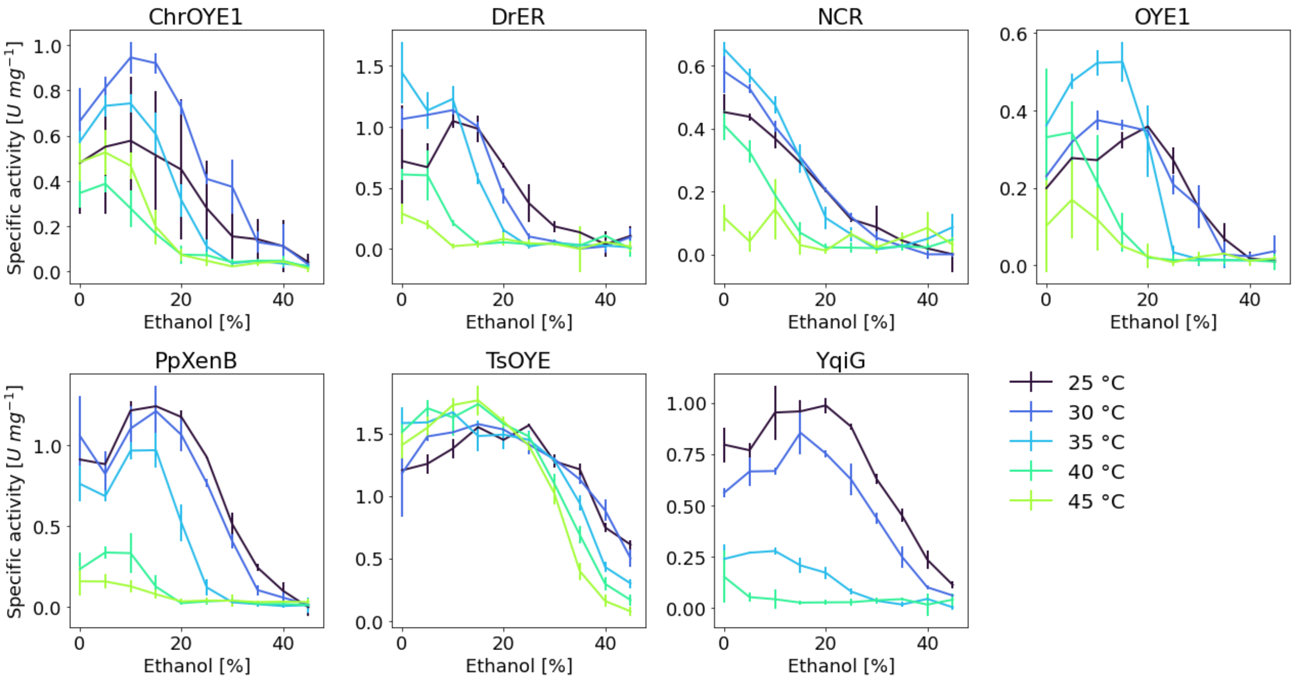

Supplementary Fig. 8 Specific activity of ene reductases in the presence of varied concentration of ethanol. Activity was measured in the presence of increasing amounts of ethanol at different reaction temperatures from 25 °C to 45 ° under following reaction conditions: 50 mM sodium phosphate buffer pH 7.4, 10 mM cyclohex-2-enone, 0.2 mM NAD(P)H. The mean activity was calculated from three replicates and the error bar marks the standard error of the mean using Gaussian error propagation.

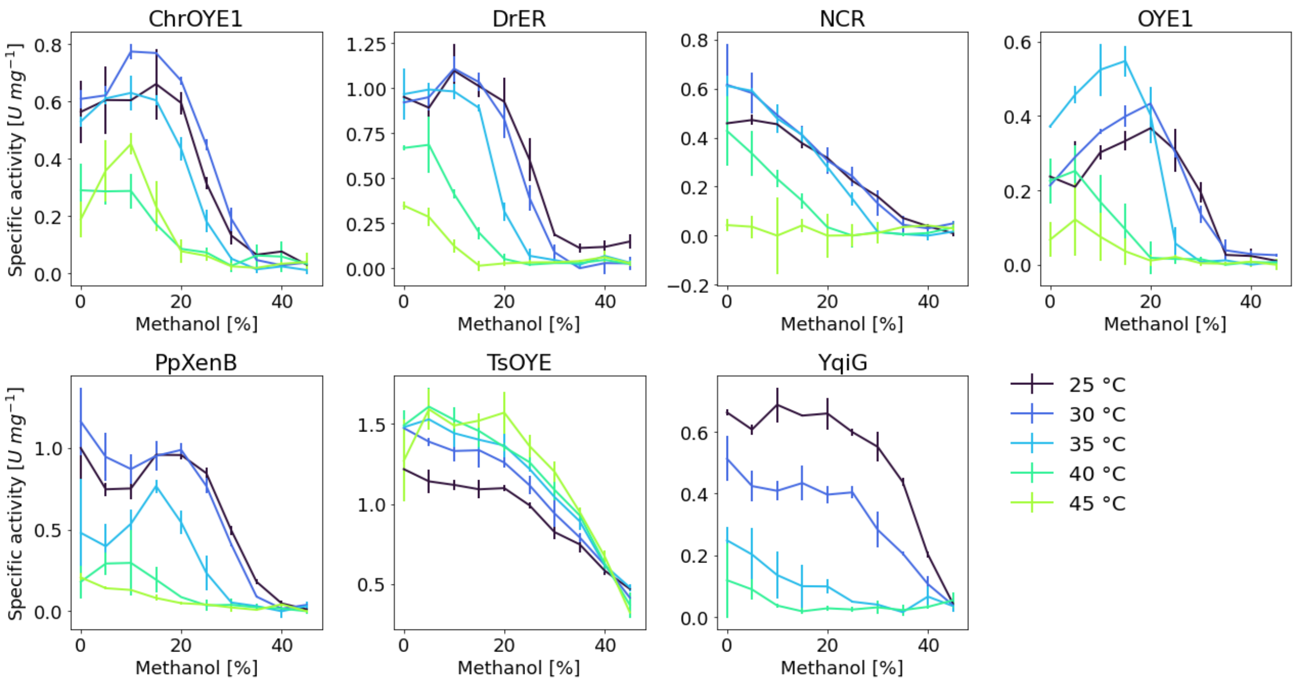

Supplementary Fig. 9 Specific activity of ene reductases in the presence of varied concentration of methanol. Activity was measured in the presence of increasing amounts of methanol at different reaction temperatures from 25 °C to 45 ° under following reaction conditions: 50 mM sodium phosphate buffer pH 7.4, 10 mM cyclohex-2-enone, 0.2 mM NAD(P)H. The mean activity was calculated from three replicates and the error bar marks the standard error of the mean using Gaussian error propagation.

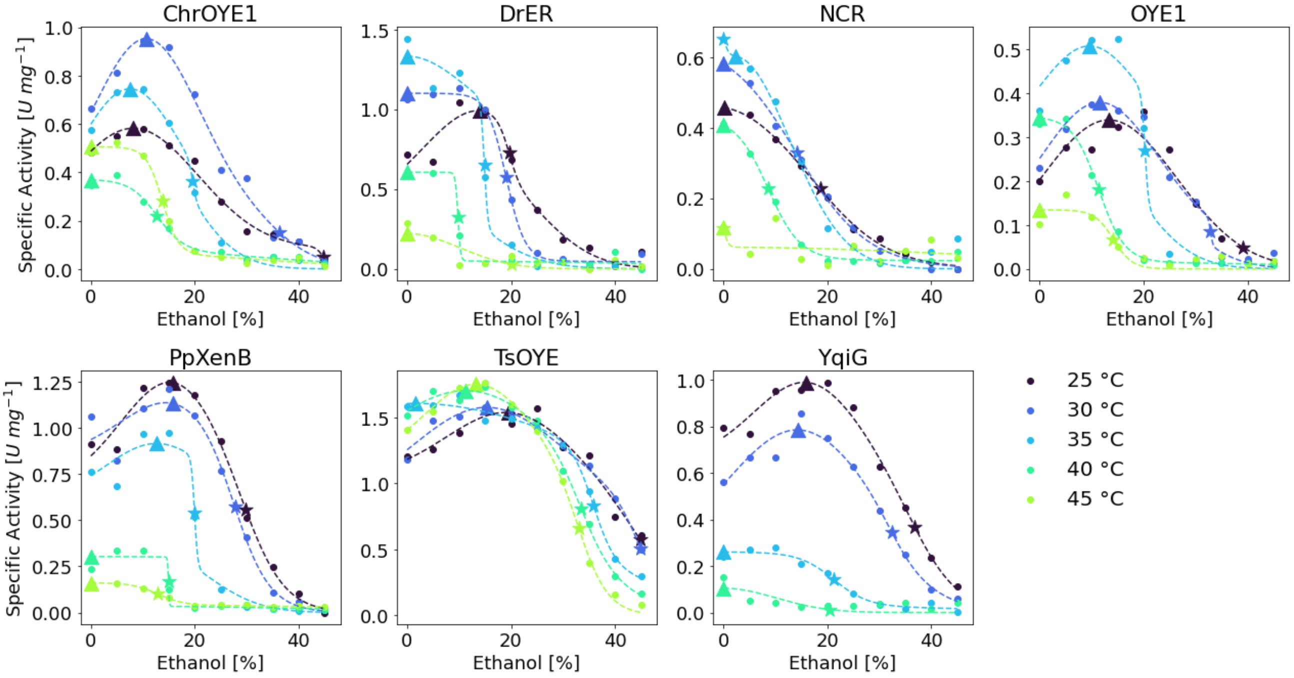

Supplementary Fig. 10 Activity-solvent model fitted to activity data of EREDs in the presence of increasing amount of ethanol. The fits are displayed as dashed lines, the measured initial activity is given as round symbols and are also shown in **Supplementary Figure 8**. The color of the curve represents the reaction temperature. The suggested model fits the data well, the star marks $c_{A_{50}}$ the concentration of largest loss in activity and the triangle marks $c_{A_{max}}$ the concentration of maximal activity.

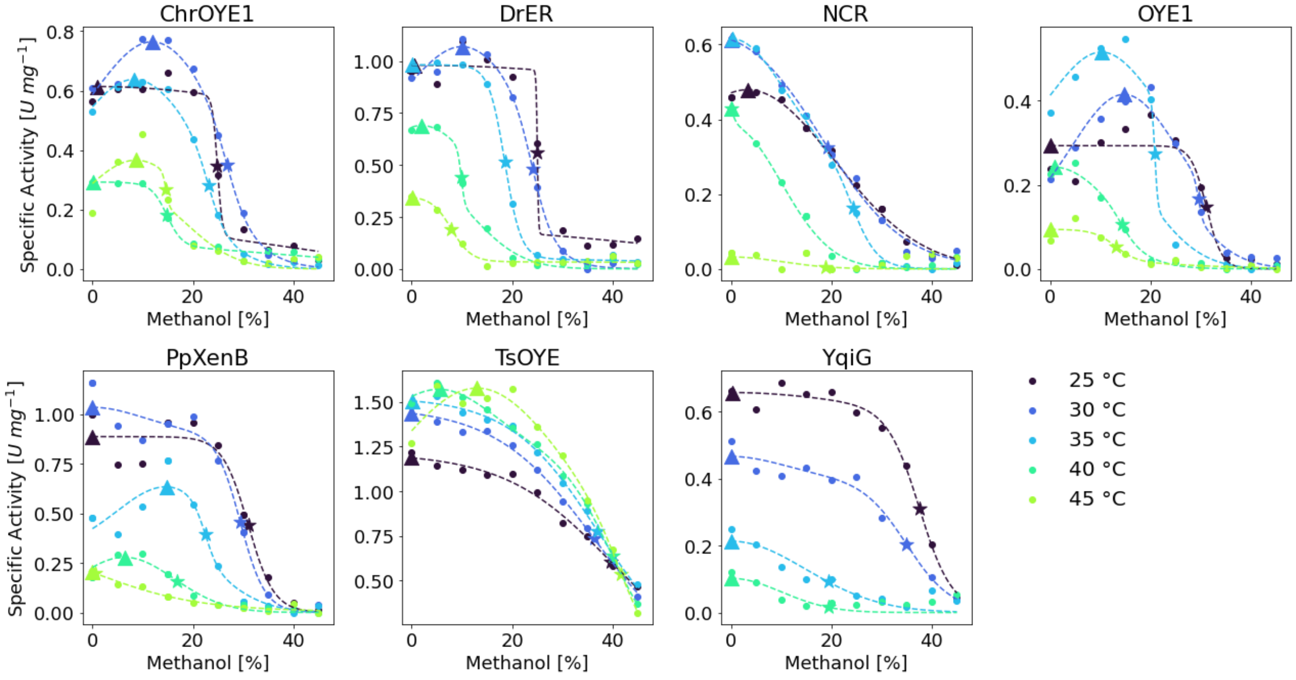

Supplementary Fig. 11 Activity-solvent model fitted to activity data of EREDs in the presence of increasing amount of methanol. The fits are displayed as dashed lines, the measured initial activity is given as round symbols and are also shown in **Supplementary Figure 9**. The color of the curve represents the reaction temperature. The suggested model fits the data well, the star marks $c_{A_{50}}$ the concentration of largest loss in activity and the triangle marks $c_{A_{max}}$ the concentration of maximal activity.

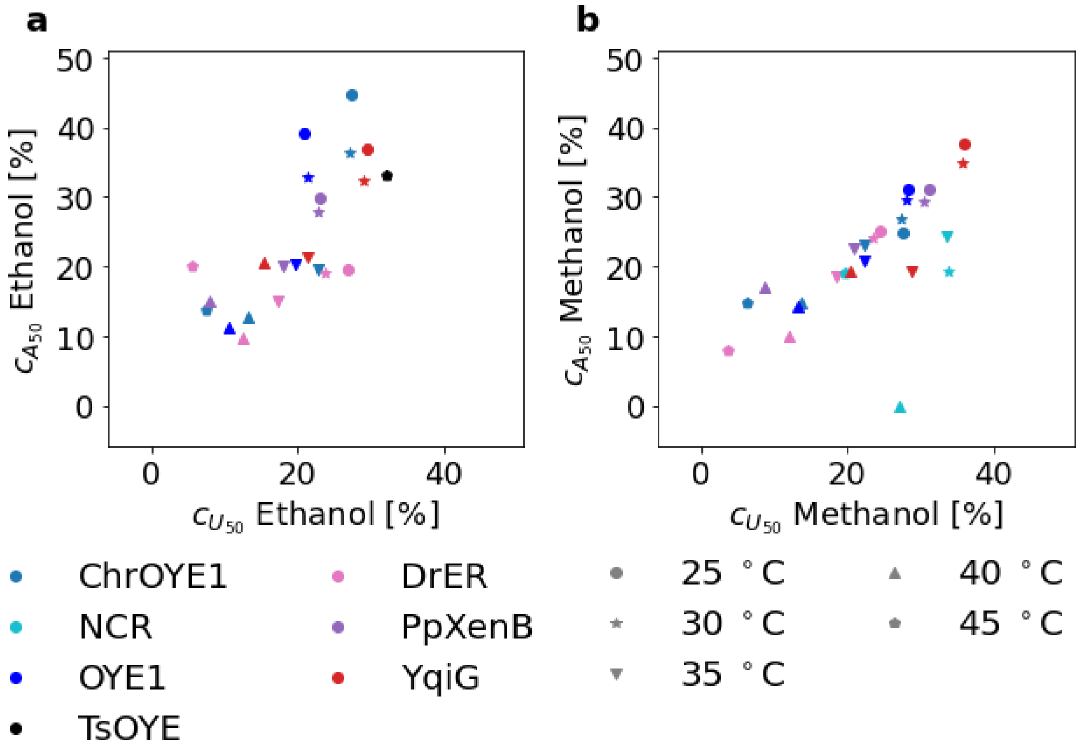

Supplementary Fig. 12 Correlation of unfolding concentration $c^T_{U_{50}}$ and inactivation concentration $c_{A_{50}}$ for (a) ethanol and (b) methanol. The plots show the values for seven EREDs where both, the unfolding and the activity data were within the measurable range and could be fitted to the two-state model of unfolding or the described activity model at the given temperatures. Thus, the transition of unfolding at the given temperature was observed between 0 and 45% (v/v) co-solvent and the activity under the same conditions could be measured. The different enzymes are indicated by color and the different reaction/experiment temperature by the shape of the symbol as given in the legend. The Pearson correlation coefficient across these enzymes and temperatures for ethanol is 0.724 and for methanol is 0.672.

Supplementary Table 3 Ranking of enzyme stability depending on mode of analysis (melting temperature and unfolding concentration) including values calculated from experimental data.

Enzyme	Stability measure				
	T_m	$T_m^{10\%}$	$c_{U_{50}}^{30\,°C}$	$T_m^{10\%}$	$c_{U_{50}}^{30\,°C}$
	[°C]	[°C]	[% (v/v)]	[°C]	[% (v/v)]
	w/o solvent	Ethanol		Methanol	
DrER	57.9	46.3	23.7	52.3	23.4
YqiG	54	45.3	29	49.6	35.8
ChrOYE1	46.7	38.3	27	40.3	27.3
OYE1	45.3	42	21.4	43	27.9
PpXenB	45.3	39.3	22.9	41.3	30.5

Note: This table shows the underlying stability values from experimental data that were translated into a ranking and are given in Table 2.

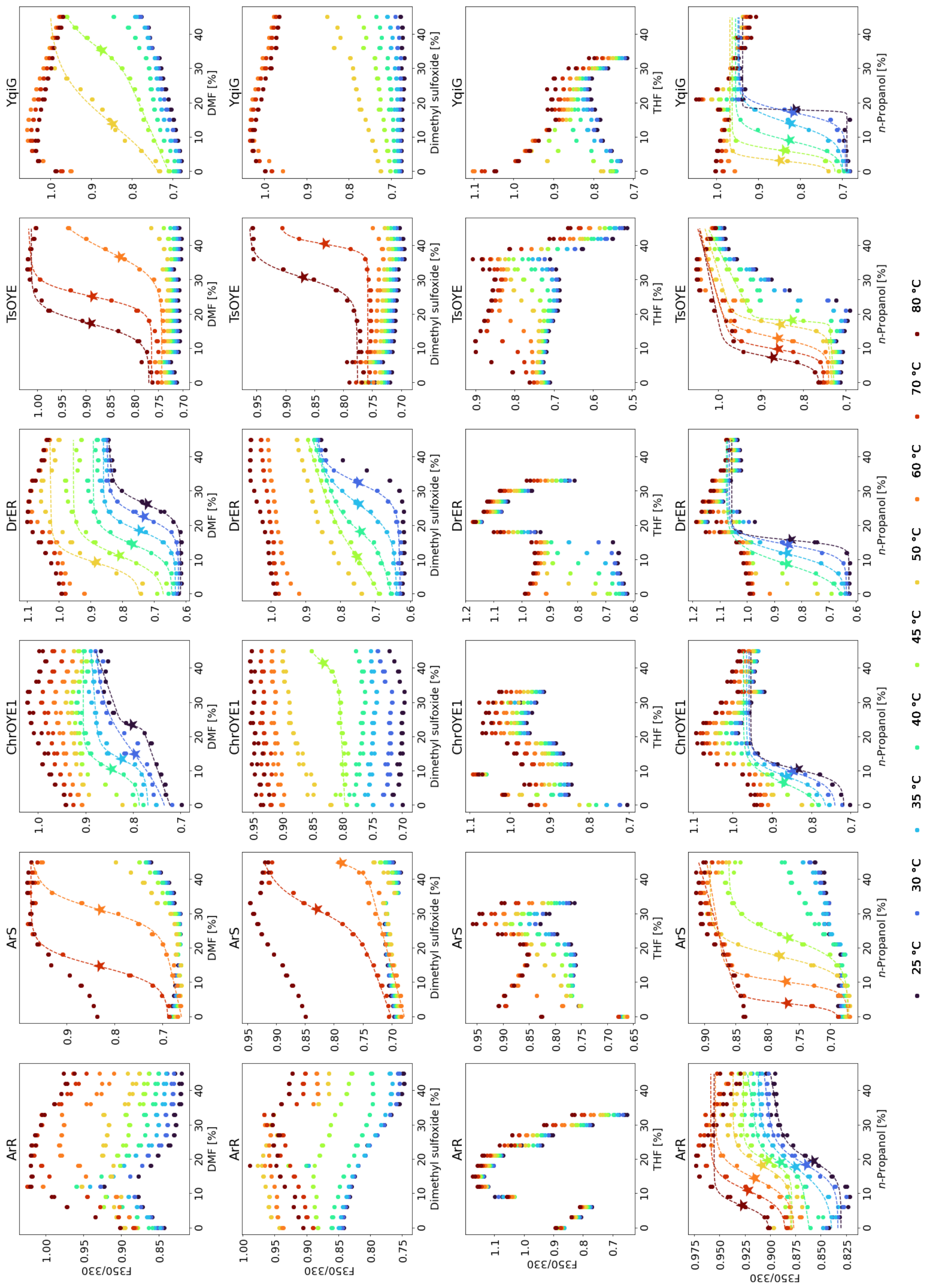

Supplementary Fig. 13 Unfolding curves for EREDS in further solvents and two transaminases. Unfolding of ene reductases and transaminases in 50 mM sodium phosphate buffer pH 7.4 supplemented with varying solvents at different temperatures measured by nanoDSF. The dashed lines show fits of the recorded data to the two-state model of unfolding (**Equation (2)**). The star marks the unfolding concentration $c_{U_{50}}$.

Supplementary Table 4 $c_{U_{50}}^{T}$ values for various EREDs in further solvents as well as $c_{U_{50}}^{T}$ values for two transaminases (ArR, ArS).

Enzyme	solvent	T [°C]	$c_{U_{50}}^{T}$ [% (v/v)]
ChrOYE1	DMF	25	23
ChrOYE1	DMF	30	15
ChrOYE1	DMF	35	13
ChrOYE1	DMF	40	11
ChrOYE1	DMSO	45	41
ChrOYE1	nprop	25	11
ChrOYE1	nprop	30	10
ChrOYE1	nprop	35	8
ChrOYE1	nprop	40	7
DrER	DMF	25	26
DrER	DMF	30	23
DrER	DMF	35	19
DrER	DMF	40	15
DrER	DMF	45	11
DrER	DMF	50	9
DrER	DMSO	30	33
DrER	DMSO	35	26
DrER	DMSO	40	18
DrER	DMSO	45	11

DrER	nprop	25	16
DrER	nprop	30	14
DrER	nprop	35	12
DrER	nprop	40	9
TsOYE	DMF	60	37
TsOYE	DMF	70	25
TsOYE	DMF	80	17
TsOYE	DMSO	70	41
TsOYE	DMSO	80	31
TsOYE	nprop	45	18
TsOYE	nprop	50	17
TsOYE	nprop	60	13
TsOYE	nprop	70	10
TsOYE	nprop	80	7
YqiG	DMF	45	35
YqiG	DMF	50	14
YqiG	nprop	25	18
YqiG	nprop	30	17
YqiG	nprop	35	14
YqiG	nprop	40	9
YqiG	nprop	45	6

YqiG	nprop	50	3
ArR	nprop	25	19
ArR	nprop	30	18
ArR	nprop	35	18
ArR	nprop	40	19
ArR	nprop	45	20
ArR	nprop	50	18
ArR	nprop	60	14
ArR	nprop	70	11
ArR	nprop	80	7
ArS	DMF	60	31
ArS	DMF	70	15
ArS	DMSO	60	45
ArS	DMSO	70	31
ArS	EtOH	50	25
ArS	EtOH	60	17
ArS	EtOH	70	6
ArS	MeOH	45	47
ArS	MeOH	50	44
ArS	MeOH	60	24
ArS	MeOH	70	9

ArS	nprop	45	23
ArS	nprop	50	18
ArS	nprop	60	10
ArS	nprop	70	4

Supplementary Note 1 The DNA sequences for the new EREDS of this study. They were ordered in pET28a(+) using restriction sites NdeI and XhoI (which are underlined) to obtain them with a cleavable N-terminal 6x His-tag.

DrER:

catATGACCGTGAGCAGTGCAGCCGCACCGCAGCCGGCAAGCCCTGCAGCACCTCT
GCTGTTCACCCCGCTGAAACTGCGTAGCCTGGAACTGCCGAATCGTGTTGTTGTT
AGTCCGATGTGTACCTATAGTGCAACCGATGGTGTGGCCAATGAATTCCATCTGG
TGCATCTGGGCCAGTATGCACTGGGCGGCGCAGGTCTGATTCTGGCAGAAGCCAC
CGCCGTTAGTCCGGAAGGCCGCATTACCCCGGAAGATCTGGGCCTGTGGGATGAT
CGTCAGATTGTTCCGCTGGGTCATATTACCGACTTCGTGCATCAGCATGGTGGCC
ATATTGGCGTGCAGCTGGCCCATGCAGGCCGTAAAGCAAGCACCTATGCACCGTG
GCGTGGCAAAGGTGCCGTTCCGGCCGAACTGGGCGGCTGGCAAGTGATTGGTCC
GGATGAAAATAGCTTCCATGATCTGTTCCCGACCCCGGCCATGATGGGCGCCGAT
GAACTGCGTGGCGTTGTGGATGCCTTCAGTGCCGCCGCACGCCGTGCCCAAGTTG
CAGGCTTCGATGCAGTTGAAGTGCATGCAGCCCATGGTTATCTGCTGCATCAGTT
CCTGAGTCCGCTGGCCAATACCCGTACCGATGATTATGGTGGTAGCTTCGAAAAT
CGTACCCGTCTGCTGCTGGAAGTTGTTCGTGCCGTTCGCCATGTGTGGCCGGCAC
ATCTGCCGCTGTTCGTGCGCCTGAGCGCCACCGATTGGGCCGAAGGTGGCTGGGA
TCTGGAACAGACCGTTCAGCTGAGCAAACTGCTGAAATATGAAGGTGTGGATGTG
CTGGATATTAGTAGCGGCGGCCTGACCGCAGCACAGCAGATTGAAGTGGGCCCG
GGTTATCAGGTGCCGTTCGCAGCCGCAGTGAGCCGTGCCGAAACCGAAATTAGCG
TTATGGCCGTTGGTCTGATTGAAACCGGCGCCCAGGCCGAAGCAATTCTGCAGGC
CGGTGATGCCGATCTGATTGCCCTGGGCCGCCCGTTCCTGCGTGATCCTCATTGGG
CACAGCGTGCCGCACGCGAACTGGGTCTGCGTCCGGTGAGCATTGATCAGTATGC
CCGCGCCGGTTGGTAActcgag

RmER

catATGCCGCATCTGTTCGATCCGTATCGTATTGGTAATCTGGAACTGGCAAATCGT
ATTGCCATTGCACCGATGTGCCAGTATAGCGCCCAGGAAGGTAATGCCACCGATT
GGCACATGATTCATCTGGGTCAGATGGCACTGAGCGGCGCCGGCCTGCTGATTAT
TGAAGCCACCGCCGTGAGCCCGGAAGGTCGTATTACCCCGACCGATCTGGGTCTG
TATAATGATGCCAATGAAGCAGCCCTGGGCCGCGTTCTGGGTGCCGTTCGTAATC
ATAGCCCGATTGCAGTTACCATTCAGCTGGCCCATGCAGGTCGCAAAGCCAGCAG
CGAAGCCCCGTGGGATGGCGGTGGCCAGATTCGTCCGGATCAGCCGCGTGGTTGG
CAGACCTTCGCCCCGAGTGCCGTGCCGCATGCCGCTGGTGAAGTGCCGCCGGCAG
CCTTAGATAAAGCAGGCATGAAAAAAATTCGTGATGACTTCGTTGCAGCCGCAAA
ACGCGCAGCCCGCCTGGGTATTGAAGGCATTGAAGTTCATGGCGCCCATGGTTAT
CTGCTGCATCAGTTCCTGAGCCCGATTGCCAATCATCGCACCGATGAATATGGTG
GCAGTCTGGAAAATCGTATGCGCTTCCCGCTGGAAGTGTTCGATGCAGTGCGCGA
AGCATTCCCGGCAGAACGCCCGGTGTGGATGCGTGTTAGTGCAACCGATTGGGTG
CCGAATGGCTGGGATATTGAAGGTACCATTGCCCTGAGTCATGAACTGAAAGCCC
GCGGCAGCGCAGCCGTTCATGTTAGTACCGGTGGCGTGAGTCCGCAGCAGGCAAT
TAAAATTGGCCCGGGTTATCAGGTTCCGTATGCCCAGCGTGTTAAAGCCGAAGTG
GGTCTGCCGACCATGGCAGTTGGTCTGATTACCGAAGCCGAACAGGCCGAAGCA
ATTATTGCCAATAATGAAGCAGATATTATCAGCATTGCACGTGCAATGCTGTATG
ATCCGCGTTGGCCGTGGCATGCAGCCGCAAAGCTGGGTGCCAGCGTTAATGCCCC

GAAACAGTATTGGCGCAGTCAGCCGCGCGGCCTGGAAAAACTGTTCAAAGATGC
ACACTTCGGCCAGCGTTAActcgag

ChrOYE1

catATGAGCACCGAAAGTCTGTTCACCCCGTTCAAATATAAAAATCTGGAACTGAA
AAACCGCATTGTGATGGCCCCGATGACCCGTGCACAGAGTGATAATGGCGTGCCG
ACCCAGCAGATTGCCGATTATTATGCACGTCGCGCCGCCGCCGAAGTTGGCTTAA
TTCTGAGTGAAGGCACCGTTATTAATCGCCCGGCAAGTAAAAATATGCAGAATAT
TCCGGACTTCTATGGCACCGAAGCACTGAATGGCTGGAAAAATGTGATTGATGCC
GTGCATCATAATGGCGGTAAAATGGGCCCGCAGATCTGGCATGTGGGTGATACCC
GCAGCACCCCGGATTATCCGCTGGAAGATATGGAAAAAGCAAGCACCATGACCC
TGGAAGATATTCAGGATACCATTGCCCAGTTCGCAGCAAGCGCCAAAAGCGCCA
AAGATCTGGGCTTCGATGTGCTGGAAATTCATGGTGCACATGGCTATCTGATTGA
TCAGTTCTTCTGGGAAGGTACCAATACCCGTACCGATGAATATGGCGGCAAAACC
ATTAAAGAACGCAGCCGCTTCGCAGTTGATGTGGTTAAAGCCATTCGCGCCGCAG
TGGGTGAAGACTTCACCATTATTATTCGTCTGAGCCAGTGGAAACAGCAGGATTA
TAGCGTGAAACTGGCACATACCCCGGAAGAAATGGAAGAATGGCTGCTGCCGCT
GAAAGATGCAGGCGTTGATATCTTCCATTGCAGTCAGCGTCGCTTCTGGGAACCG
GAATTCGAAGGTAGCGATCTGAACTTCGCAGGTTGGGCAAAAAAAATTACCGGC
CAGCCGACCATTACCGTGGGCAGCGTTGGTCTGGAAGGCGACTTCATGGCCGCCT
TCGGCGGCCAGGGCACCGAAAAAGCAGATCTGACCGAACTGACCAAACGTCTGG
AACGCGGTGACTTCGATCTGGTTGCAGTTGGTCGTGCACTGCTGCAGGATCCGGA
ATGGGCAAAAAAGGTTAAAGAACAGAATACCGAAGCATTACTGGACTTCAGTGC
CGAAAGCCTGGGTGTGCTGTATTAActcgag

LacER

catATGAGCGGCTATCACTTCCTGAAACCGTTCACCTTCAAACATCAGACCATTACC
CTGAAAAATCGCATTGTTATTCCGCCGATGACCACCCGTCTGAGCTTCGAAGATG
GCACCGTGACCCGCGATGAAATTCGTTATTATCAGCAGCGCGCAGGCGGCGTGGG
TATGTTCATTACCGGCACCGCAAATGTTAATGCCCTGGGTAAAGGCTTCGAAGGT
GAACTGAGCGTGGCCGATGATCGCTTCATTCCGGGTCTGAGCAAACTGGCAGCCG
CCATGAAAACCGGTGGCACCAAAGCCATTCTGCAGATCTTCAGTGCCGGTCGTAT
GAGCAATAGCAAAATTCTGCGCGGTGAACAGCCGGTTAGCGCCAGTGCAGTTGC
CGCCCCGCGTGCTGGTTATGAAACACCTCGTGCCCTGACCAGCGCCGAAATTGAA
GCAACCATTCATGACTTCGGCCAGGCCGTTCGTCGCGCAATTCTGGCAGGCTTCG
ATGGCATTGAACTGCATGGCGCAAATACCTATCTGATTCAGCAGTTCTATAGCCC
GAATAGTAATCGTCGTACCGATGAATGGGGTGGTGATCGTGATAAACGCATGCGC
TTCCCGCTGGCAGTTGTTCATGAAGCAGAAAAAGTGATTGCCACCATTGCAGATC
GCCCGTTCCTGCTGGGCTATCGCATTAGTCCGGAAGAACTGGAACAGCCGGGTAT
TACCCTGGATGATACCCTGGCCCTGATTGATGCACTGAAACAGACCAAAATTGAT
TATCTGCATGTGAGTCAGAGCGATGTGTGGCGCACCAGCCTGCGTAATCCGGAAG
ATACCGCAATTATGAATGAACAGATTCGTGATCATGTGGCAGGTGCCTTCCCGGT
GATTGTTGTGGGTGGTATTAAAACACCTGCAGATGCCGAAAAAGCCGCAGAAAG
CTTCGATCTGGTTGCCATTGGTCATGAAATGATTCGCGAACCGCATTGGGTGCAG
AAAGTTCTGGATCATGATGAAAAAGCCATTCGCTATCAGATTGCACCGGCAGATC
TGGAAGAACTGGGCATTGCCCCGACCTTCCTGGACTTCATTGAAAGTATTAGCGG

CGGTGCCAAAGGTGTGCCGCTGACCACCGCCCAGAGTGTGACCAGCAGCAATGTT
ACCCAGGATTAActcgag

PpXenB

catATGACCACCCTGTTCGATCCGATTAAACTGGGCGATCTGCAGCTGCCGAATCG
CATTATTATGGCACCGCTGACCCGTTGCCGCGCAGATGAAGGCCGCGTTCCGAAT
GCACTGATGGCAGAATATTATGTGCAGCGTGCCAGTGCCGGTCTGATTCTGAGTG
AAGCCACCAGCGTGAGCCCGATGGGTGTTGGTTATCCGGATACCCCGGGCATCTG
GAATGATGAACAGGTGCGCGGCTGGAATAATGTTACCAAAGCCGTTCATGCAGC
CGGTGGTCGCATCTTCCTGCAGCTGTGGCATGTTGGTCGCATTAGTCATCCGAGCT
ATCTGAATGGCGAACTGCCGGTTGCACCGAGTGCAATTCAGCCGAAAGGCCATGT
TAGCCTGGTTCGTCCGCTGAGTGATTATCCGACCCCGCGTGCACTGGAAACCGAA
GAAATTAATGATATTGTTGAGGCATACCGCAGTGGTGCCGAAAATGCAAAAGCC
GCCGGCTTCGATGGCGTTGAAATTCATGGCGCAAATGGCTATCTGCTGGATCAGT
TCCTGCAGAGCAGCACCAATCAGCGTACCGATCGCTATGGTGGTAGCCTGGAAAA
TCGCGCACGTCTGCTGCTGGAAGTGACCGATGCCGCAATTGAAGTGTGGGGTGCA
CAGCGCGTTGGCGTTCATCTGGCACCGCGCGCCGATGCACATGATATGGGCGATG
CCGATCGCGCAGAAACCTTCACCTATGTGGCCCGTGAACTGGGCAAACGTGGTAT
TGCCTTCATCTGTAGTCGTGAACGTGAAGCAGATGATAGCATTGGTCCGCTGATT
AAAGAAGCATTCGGCGGCCCGTATATTGTTAATGAACGCTTCGATAAAGCCAGTG
CAAATGCCGCCCTGGCAAGTGGTAAAGCCGATGCAGTTGCATTCGGTGTTCCGTT
CATTGCAAATCCGGATCTGCCGGCACGTCTGGCCGCAGATGCACCGCTGAATGAA
GCACATCCGGAAACCTTCTATGGCAAAGGTCCGGTGGGCTATATTGATTATCCGC
GTCTGTAActcgag

TsOYE

catATGGCACTGCTGTTCACCCCGCTGGAACTGGGTGGTCTGCGTCTGAAAAATCG
CCTGGCCATGAGTCCGATGTGCCAGTATAGCGCCACCCTGGAAGGTGAAGTGACC
GATTGGCATCTGCTGCATTATCCGACCCGTGCCCTGGGCGGTGTTGGTCTGATTCT
GGTGGAAGCAACCGCAGTGGAACCGCTGGGTCGCATTAGCCCGTATGATCTGGG
CATCTGGAGTGAAGATCATCTGCCGGGTCTGAAAGAACTGGCCCGCCGTATTCGT
GAAGCCGGCGCCGTGCCGGGCATTCAGCTGGCACATGCAGGCCGCAAAGCAGGT
ACCGCCCGTCCGTGGGAAGGCGGTAAACCGCTGGGTTGGCGTGTGGTTGGCCCGA
GTCCGATTCCGTTCGATGAAGGTTATCCGGTTCCGGAACCGCTGGATGAAGCAGG
TATGGAACGTATTCTGCAGGCCTTCGTTGAAGGTGCACGCCGCGCACTGCGCGCA
GGCTTCCAAGTTATTGAACTGCACATGGCACATGGTTATCTGCTGAGCAGCTTCCT
GAGCCCGCTGAGTAATCAGCGTACCGATGCATACGGTGGCAGCCTGGAAAATCG
TATGCGCTTCCCGCTGCAGGTTGCCCAGGCAGTGCGCGAAGTTGTGCCGCGCGAA
CTGCCGCTGTTCGTGCGCGTTAGTGCAACCGATTGGGGCGAAGGTGGTTGGAGTC
TGGAAGATACCCTGGCATTCGCACGTCGTCTGAAAGAATTAGGCGTGGATCTGCT
GGATTGCAGCAGCGGTGGTGTTGTTCTGCGCGTTCGTATTCCGCTGGCACCGGGC
TTCCAGGTGCCGTTCGCAGATGCAGTTCGTAAACGTGTTGGCCTGCGTACCGGCG
CCGTGGGTCTGATTACCACCCCGGAACAGGCCGAAACCTTACTGCAGGCAGGCA
GTGCCGATCTGGTTCTGCTGGGCCGTGTTCTGCTGCGCGATCCGTACTTCCCGCTG
CGTGCAGCCAAAGCACTGGGTGTTGCCCCGGAAGTTCCGCCGCAGTATCAGCGTG
GCTTCTAActcgag

YqiG

catATGAACCCGAAATATAAGCCGCTGTTCGAACCGTTCACCTTCAAAAGTGGTGT
GACCATTAATAATCGTATTGCCGTGGCCCCGATGACCCATTATGCCAGCAATGAA
GATGGCACCATTAGTGAAGCCGAACTGGATTATATTATTCCGCGCAGCAAAGAAA
TGGGCATGGTGATTACCGCATGCGCAAATGTGACCCCGGATGGCAAAGCCTTCCC
GGGCCAGCCGGCAATTCATGATGATAGTAATATTCCGGGCCTGAAAAAACTGGC
ACAGGCCATTCAGGCACAGGGTGCCAAAGCAGTGGTTCAGATTCATCATGGTGGC
ATTGAATGCCCGAGCGAACTGGTTCCGCAGCAGGATGTGGTGGGTCCGAGTGATG
TGTTCGATAATGGCAAACAGATTGCCCGTGCACTGACCGAAGAAGAAGTGGAAA
ATATTGTGAAAGCCTTCGGTGAAGCCACCCGTCGCGCCATTGAAGCAGGCTTCGA
TGGTGTTGAAATTCATGGTGCAAATGGCTATCTGATTCAGCAGTTCTATAGCCCG
AAAACCAATCAGCGCACCGATCGTTGGGGTGGCAGTGATGAAAAACGCCTGGCC
TTCCCGCTGGCAATTGTGGATGAAGTTAAAAAAGCCGCAAGTGAACATGCAAAA
GGCGCCTTCCTGGTGGGCTATCGTCTGAGCCCGGAAGAACCGGAAACACCTGGTC
TGACCATGACCGAAACCTATACCCTGGTTGATGCACTGGGCGATAAAGAACTGGA
TTACCTGCATATTAGCCTGATGGATGTGAATAGCAAAGCCCGTCGCGGTGCCGAT
CCGACCCGCACCAGAATGGATCTGCTGAATGAACGCGTTGGTAATAAAGTTCCGC
TGATTGCCGTTGGCAGTATTCATAGTGCCGATGATGCCCTGGCCGTTATTGAAAA
TGGCATTCCGCTGGTTGCAATGGGTCGCGAAATTCTGGTGGATCCGAATTGGACC
GTGAAAGTGAAAGAAGGTCGTGAAAAACAGATTGAAACCGTTATTAAAGGTACC
GATAAAGAAAAATACCACCTGCCGGAACCGCTGTGGCAGGCCATTGTTAATACCC
AGGGTTGGGTTCCGTATAAAGATTAActcgag

Supplementary Table 5 Raw melting temperature and activity data

Enzyme	Solvent	Concentration	Tm_mean[°C]	Tm_std[°C]	Activity_mean[U mg^{-1}]	Activity_std[U mg^{-1}]
ChrOYE1	DMSO	0	47.00	0.00	1.03	0.03
ChrOYE1	DMSO	5	45.67	0.58	1.19	0.01
ChrOYE1	DMSO	10	44.33	0.58	1.23	0.06
ChrOYE1	DMSO	15	43.67	0.58	1.21	0.02
ChrOYE1	DMSO	20	43.00	0.00	1.12	0.04
ChrOYE1	DMSO	25	42.00	0.00	0.95	0.03
ChrOYE1	DMSO	30	40.00	1.00	0.72	0.04
ChrOYE1	EtOH	0	47.00	0.00	1.06	0.06
ChrOYE1	EtOH	5	44.00	0.00	1.16	0.02
ChrOYE1	EtOH	10	38.33	1.15	1.23	0.03
ChrOYE1	EtOH	15	35.67	1.53	1.32	0.02
ChrOYE1	EtOH	20	34.00	1.00	1.21	0.03
ChrOYE1	EtOH	25	32.67	0.58	0.95	0.03
ChrOYE1	EtOH	30	30.00	0.00	0.63	0.11
ChrOYE1	MeOH	0	46.67	0.58	1.11	0.04
ChrOYE1	MeOH	5	46.00	1.00	1.13	0.05
ChrOYE1	MeOH	10	40.33	1.53	1.09	0.03
ChrOYE1	MeOH	15	36.00	0.00	1.14	0.13
ChrOYE1	MeOH	20	37.00	1.00	1.10	0.02
ChrOYE1	MeOH	25	35.33	0.58	0.92	0.04
ChrOYE1	MeOH	30	31.33	1.15	0.45	0.11
ChrOYE1	iprop	0	46.33	0.58	1.14	0.15
ChrOYE1	iprop	5	41.67	0.58	1.25	0.15
ChrOYE1	iprop	10	36.67	1.15	1.35	0.05
ChrOYE1	iprop	15	35.00	0.00	1.26	0.15
ChrOYE1	iprop	20	33.00	0.00	1.11	0.03
ChrOYE1	iprop	25	30.00	0.00	0.95	0.07
ChrOYE1	iprop	30	29.00	0.00	0.83	0.11
ChrOYE1	nprop	0	46.67	0.58	1.00	0.22
ChrOYE1	nprop	5	39.33	0.58	1.18	0.05
ChrOYE1	nprop	10	32.33	0.58	1.10	0.12
ChrOYE1	nprop	15	29.67	0.58	0.97	0.05
ChrOYE1	nprop	20	28.33	0.58	0.66	0.11
ChrOYE1	nprop	25	27.00	0.00	0.52	0.10
ChrOYE1	nprop	30	27.00	0.00	0.41	0.08
DrER	DMSO	0	58.00	0.00	1.49	0.15
DrER	DMSO	5	55.00	0.00	1.57	0.14
DrER	DMSO	10	53.33	0.58	1.55	0.05
DrER	DMSO	15	53.00	1.00	1.48	0.08
DrER	DMSO	20	52.00	0.00	1.22	0.08
DrER	DMSO	25	51.67	0.58	1.03	0.08
DrER	DMSO	30	52.00	0.00	0.71	0.05
DrER	EtOH	0	57.67	0.58	1.56	0.05
DrER	EtOH	5	53.00	1.00	1.31	0.10

DrER	EtOH	10	46.33	1.53	1.38	0.20
DrER	EtOH	15	42.67	0.58	1.40	0.06
DrER	EtOH	20	40.67	0.58	1.28	0.03
DrER	EtOH	25	34.33	1.15	0.84	0.07
DrER	EtOH	30	35.67	1.53	0.42	0.03
DrER	MeOH	0	58.00	0.00	1.42	0.05
DrER	MeOH	5	57.00	1.00	1.30	0.05
DrER	MeOH	10	52.33	0.58	1.22	0.05
DrER	MeOH	15	49.67	1.53	1.16	0.07
DrER	MeOH	20	45.00	1.73	1.10	0.01
DrER	MeOH	25	41.00	2.00	0.81	0.06
DrER	MeOH	30	38.33	3.06	0.39	0.15
DrER	iprop	0	58.00	0.00	1.56	0.09
DrER	iprop	5	49.00	3.00	1.47	0.06
DrER	iprop	10	43.67	4.04	1.54	0.10
DrER	iprop	15	38.33	2.08	1.41	0.03
DrER	iprop	20	35.67	1.53	1.16	0.04
DrER	iprop	25	32.67	1.53	0.81	0.15
DrER	iprop	30	28.67	1.15	0.12	0.01
DrER	nprop	0	58.00	0.00	1.34	0.11
DrER	nprop	5	47.00	7.21	1.31	0.07
DrER	nprop	10	36.00	1.73	1.32	0.12
DrER	nprop	15	31.33	0.58	1.03	0.28
DrER	nprop	20	31.00	1.00	0.24	0.05
DrER	nprop	25	28.67	0.58	0.04	0.02
DrER	nprop	30	27.00	0.00	0.02	0.01
LacER	DMSO	0	50.67	0.58	12.50	0.46
LacER	DMSO	5	49.67	0.58	13.07	0.34
LacER	DMSO	10	49.33	0.58	12.77	0.49
LacER	DMSO	15	48.67	0.58	12.67	0.90
LacER	DMSO	20	48.00	0.00	11.04	0.59
LacER	DMSO	25	46.67	0.58	10.15	0.24
LacER	DMSO	30	45.00	1.00	8.28	0.29
LacER	EtOH	0	51.00	0.00	12.36	0.55
LacER	EtOH	5	47.67	0.58	12.68	0.45
LacER	EtOH	10	42.33	1.53	11.36	0.58
LacER	EtOH	15	37.67	1.15	11.85	0.69
LacER	EtOH	20	36.00	0.00	13.07	0.49
LacER	EtOH	25	32.67	0.58	11.56	0.42
LacER	EtOH	30	33.00	1.00	10.19	0.68
LacER	MeOH	0	52.00	0.00	20.28	1.29
LacER	MeOH	5	47.67	1.53	18.11	0.39
LacER	MeOH	10	41.00	2.00	19.52	1.76
LacER	MeOH	15	39.33	1.53	17.67	0.90
LacER	MeOH	20	35.00	1.73	15.83	1.53
LacER	MeOH	25	35.00	1.00	13.87	0.90
LacER	MeOH	30	31.33	0.58	11.13	0.93

LacER	iprop	0	51.00	0.00	17.01	1.00
LacER	iprop	5	42.67	3.06	17.15	0.99
LacER	iprop	10	41.67	0.58	19.69	1.45
LacER	iprop	15	36.33	1.15	18.37	0.93
LacER	iprop	20	35.00	1.73	16.35	0.97
LacER	iprop	25	32.33	2.52	14.39	2.30
LacER	iprop	30	31.00	1.00	11.66	0.80
LacER	nprop	0	50.33	1.15	18.41	0.74
LacER	nprop	5	39.33	0.58	18.10	0.91
LacER	nprop	10	30.33	2.08	17.83	0.65
LacER	nprop	15	28.33	0.58	15.42	0.57
LacER	nprop	20	25.00	2.65	11.85	0.66
LacER	nprop	25	26.00	0.00	7.48	0.26
LacER	nprop	30	23.00	3.00	4.60	1.28
NCR	DMSO	0	50.67	0.58	0.47	0.02
NCR	DMSO	5	49.00	0.00	0.39	0.01
NCR	DMSO	10	47.00	0.00	0.30	0.01
NCR	DMSO	15	45.33	0.58	0.23	0.01
NCR	DMSO	20	43.33	0.58	0.15	0.00
NCR	DMSO	25	42.67	0.58	0.10	0.01
NCR	DMSO	30	41.33	2.08	0.05	0.00
NCR	EtOH	0	50.00	1.73	0.47	0.02
NCR	EtOH	5	47.67	1.15	0.39	0.02
NCR	EtOH	10	43.33	0.58	0.36	0.02
NCR	EtOH	15	40.33	0.58	0.28	0.02
NCR	EtOH	20	38.00	1.00	0.21	0.00
NCR	EtOH	25	35.67	0.58	0.15	0.01
NCR	EtOH	30	34.33	0.58	0.09	0.03
NCR	MeOH	0	50.33	0.58	0.46	0.03
NCR	MeOH	5	49.00	1.00	0.43	0.01
NCR	MeOH	10	47.33	0.58	0.37	0.01
NCR	MeOH	15	45.67	0.58	0.33	0.02
NCR	MeOH	20	42.33	0.58	0.28	0.01
NCR	MeOH	25	40.00	0.00	0.22	0.03
NCR	MeOH	30	39.00	1.00	0.18	0.01
NCR	iprop	0	51.00	0.00	0.44	0.02
NCR	iprop	5	47.33	0.58	0.37	0.02
NCR	iprop	10	43.33	0.58	0.34	0.01
NCR	iprop	15	39.67	1.15	0.25	0.02
NCR	iprop	20	35.67	1.15	0.17	0.01
NCR	iprop	25	33.00	1.00	0.14	0.02
NCR	iprop	30	30.00	0.00	0.13	0.01
NCR	nprop	0	50.67	0.58	0.45	0.01
NCR	nprop	5	44.33	1.15	0.31	0.01
NCR	nprop	10	37.33	1.15	0.20	0.02
NCR	nprop	15	33.33	1.15	0.14	0.01
NCR	nprop	20	31.33	0.58	0.10	0.00

NCR	nprop	25	30.33	0.58	0.07	0.01
NCR	nprop	30	30.00	0.00	0.06	0.02
NerA	DMSO	0	40.67	0.58	0.04	0.00
NerA	DMSO	5	39.00	0.00	0.03	0.00
NerA	DMSO	10	38.33	0.58	0.02	0.00
NerA	DMSO	15	37.33	0.58	0.02	0.00
NerA	DMSO	20	37.00	0.00	0.01	0.00
NerA	DMSO	25	35.67	0.58	0.01	0.00
NerA	DMSO	30	34.67	0.58	0.00	0.00
NerA	EtOH	0	41.00	0.00	0.04	0.00
NerA	EtOH	5	38.00	0.00	0.03	0.00
NerA	EtOH	10	35.33	0.58	0.03	0.00
NerA	EtOH	15	33.33	0.58	0.02	0.00
NerA	EtOH	20	31.33	0.58	0.01	0.00
NerA	EtOH	25	30.00	0.00	0.00	0.00
NerA	EtOH	30	28.67	0.58	0.00	0.00
NerA	MeOH	0	41.00	0.00	0.04	0.00
NerA	MeOH	5	39.33	0.58	0.04	0.00
NerA	MeOH	10	37.33	1.15	0.04	0.00
NerA	MeOH	15	36.00	1.00	0.04	0.00
NerA	MeOH	20	33.33	0.58	0.03	0.00
NerA	MeOH	25	31.67	0.58	0.02	0.00
NerA	MeOH	30	30.67	0.58	0.00	0.00
NerA	iprop	0	40.67	0.58	0.03	0.00
NerA	iprop	5	37.00	1.00	0.03	0.00
NerA	iprop	10	32.67	0.58	0.03	0.00
NerA	iprop	15	29.67	0.58	0.02	0.00
NerA	iprop	20	27.67	0.58	0.01	0.00
NerA	iprop	25	24.33	3.79	0.01	0.00
NerA	iprop	30	22.33	0.58	0.00	0.01
NerA	nprop	0	40.33	0.58	0.03	0.00
NerA	nprop	5	34.33	0.58	0.02	0.00
NerA	nprop	10	28.33	0.58	0.02	0.00
NerA	nprop	15	22.67	3.06	0.01	0.01
NerA	nprop	20	22.67	2.31	0.00	0.01
NerA	nprop	25	21.67	2.08	0.00	0.01
NerA	nprop	30	20.67	0.58	0.00	0.00
OYE1	DMSO	0	45.00	0.00	1.31	0.12
OYE1	DMSO	5	44.33	0.58	1.43	0.16
OYE1	DMSO	10	43.33	0.58	1.35	0.05
OYE1	DMSO	15	42.00	0.00	1.31	0.14
OYE1	DMSO	20	40.67	0.58	1.38	0.06
OYE1	DMSO	25	39.33	0.58	1.32	0.13
OYE1	DMSO	30	37.33	0.58	1.32	0.08
OYE1	EtOH	0	45.33	0.58	1.49	0.06
OYE1	EtOH	5	43.33	0.58	1.58	0.11
OYE1	EtOH	10	42.00	1.00	1.78	0.10

OYE1	EtOH	15	39.67	0.58	1.97	0.14
OYE1	EtOH	20	37.67	0.58	2.00	0.17
OYE1	EtOH	25	35.33	1.15	2.10	0.04
OYE1	EtOH	30	34.33	0.58	2.46	0.11
OYE1	MeOH	0	46.00	0.00	1.55	0.06
OYE1	MeOH	5	45.00	1.00	1.72	0.05
OYE1	MeOH	10	43.00	1.00	1.81	0.07
OYE1	MeOH	15	42.00	1.00	2.07	0.07
OYE1	MeOH	20	40.33	0.58	2.23	0.15
OYE1	MeOH	25	37.67	1.15	2.42	0.11
OYE1	MeOH	30	34.67	2.08	2.58	0.11
OYE1	iprop	0	45.00	0.00	1.22	0.04
OYE1	iprop	5	44.00	1.00	1.54	0.08
OYE1	iprop	10	41.33	2.08	1.70	0.08
OYE1	iprop	15	37.33	0.58	1.86	0.06
OYE1	iprop	20	34.67	0.58	1.80	0.10
OYE1	iprop	25	33.67	0.58	2.00	0.09
OYE1	iprop	30	28.33	2.52	2.05	0.07
OYE1	nprop	0	45.00	0.00	1.30	0.03
OYE1	nprop	5	40.33	0.58	1.59	0.06
OYE1	nprop	10	34.67	0.58	1.83	0.20
OYE1	nprop	15	31.67	0.58	1.81	0.18
OYE1	nprop	20	31.00	0.00	1.86	0.15
OYE1	nprop	25	30.33	0.58	1.84	0.13
OYE1	nprop	30	27.33	1.15	1.96	0.14
PpXenB	DMSO	0	45.00	0.00	3.16	0.14
PpXenB	DMSO	5	42.33	0.58	2.99	0.08
PpXenB	DMSO	10	41.67	0.58	2.70	0.17
PpXenB	DMSO	15	40.00	1.00	2.21	0.10
PpXenB	DMSO	20	39.00	0.00	1.72	0.06
PpXenB	DMSO	25	38.33	0.58	1.31	0.34
PpXenB	DMSO	30	36.00	0.00	1.14	0.09
PpXenB	EtOH	0	45.00	0.00	4.71	0.13
PpXenB	EtOH	5	42.00	2.00	4.68	0.20
PpXenB	EtOH	10	39.33	0.58	5.04	0.44
PpXenB	EtOH	15	37.33	0.58	5.08	0.40
PpXenB	EtOH	20	36.00	0.00	5.16	0.35
PpXenB	EtOH	25	35.33	0.58	4.59	0.46
PpXenB	EtOH	30	33.33	0.58	4.05	0.27
PpXenB	MeOH	0	46.00	0.00	4.28	0.23
PpXenB	MeOH	5	43.33	2.08	3.87	0.53
PpXenB	MeOH	10	41.33	1.15	4.45	0.14
PpXenB	MeOH	15	40.33	0.58	4.08	0.13
PpXenB	MeOH	20	39.33	0.58	3.96	0.30
PpXenB	MeOH	25	35.33	2.31	3.66	0.06
PpXenB	MeOH	30	36.67	0.58	2.80	0.23
PpXenB	iprop	0	45.00	0.00	4.07	0.12

PpXenB	iprop	5	41.00	2.00	4.68	0.17
PpXenB	iprop	10	38.67	2.08	4.38	0.15
PpXenB	iprop	15	34.00	2.65	4.67	0.24
PpXenB	iprop	20	32.33	0.58	4.23	0.28
PpXenB	iprop	25	30.33	1.15	3.72	0.29
PpXenB	iprop	30	30.33	1.53	2.73	0.29
PpXenB	nprop	0	45.67	0.58	3.57	0.26
PpXenB	nprop	5	38.67	0.58	4.17	0.10
PpXenB	nprop	10	34.00	1.00	4.12	0.35
PpXenB	nprop	15	31.67	0.58	3.63	0.18
PpXenB	nprop	20	30.67	0.58	2.92	0.13
PpXenB	nprop	25	30.67	0.58	2.04	0.31
PpXenB	nprop	30	29.00	1.00	1.55	0.15
RmER	DMSO	0	51.67	0.58	1.38	0.07
RmER	DMSO	5	48.33	0.58	1.72	0.04
RmER	DMSO	10	46.00	0.00	1.66	0.02
RmER	DMSO	15	43.67	0.58	1.73	0.08
RmER	DMSO	20	41.00	1.00	1.65	0.12
RmER	DMSO	25	39.67	0.58	1.50	0.11
RmER	DMSO	30	38.00	1.00	0.94	0.10
RmER	EtOH	0	51.33	0.58	1.88	0.02
RmER	EtOH	5	47.67	1.15	1.81	0.13
RmER	EtOH	10	41.33	2.08	1.74	0.05
RmER	EtOH	15	39.33	0.58	1.68	0.15
RmER	EtOH	20	36.00	1.00	1.55	0.06
RmER	EtOH	25	33.67	1.15	1.19	0.15
RmER	EtOH	30	30.00	1.00	0.22	0.05
RmER	MeOH	0	51.33	0.58	1.72	0.02
RmER	MeOH	5	48.33	0.58	1.76	0.05
RmER	MeOH	10	45.33	1.15	1.76	0.10
RmER	MeOH	15	42.33	0.58	1.72	0.02
RmER	MeOH	20	39.67	0.58	1.65	0.06
RmER	MeOH	25	36.67	0.58	1.52	0.14
RmER	MeOH	30	33.33	0.58	1.39	0.16
RmER	iprop	0	51.00	0.00	1.33	0.03
RmER	iprop	5	44.33	1.15	1.31	0.10
RmER	iprop	10	38.33	2.31	1.36	0.04
RmER	iprop	15	36.67	0.58	1.28	0.04
RmER	iprop	20	33.33	1.15	1.16	0.08
RmER	iprop	25	28.67	2.08	0.64	0.26
RmER	iprop	30	24.00	4.00	0.03	0.19
RmER	nprop	0	51.67	0.58	1.32	0.04
RmER	nprop	5	43.67	0.58	1.39	0.04
RmER	nprop	10	34.33	0.58	1.23	0.08
RmER	nprop	15	28.33	1.53	0.82	0.12
RmER	nprop	20	27.67	0.58	0.00	0.03
RmER	nprop	25	22.67	2.08	0.01	0.04

RmER	nprop	30	27.33	1.15	0.03	0.00
TsOYE	DMSO	0	92.00	3.00	1.40	0.19
TsOYE	DMSO	5	89.00	0.00	1.92	0.25
TsOYE	DMSO	10	86.67	0.58	2.35	0.05
TsOYE	DMSO	15	84.00	0.00	2.51	0.17
TsOYE	DMSO	20	82.00	0.00	2.32	0.20
TsOYE	DMSO	25	77.67	1.53	1.90	0.17
TsOYE	DMSO	30	77.33	0.58	1.97	0.21
TsOYE	EtOH	0	92.00	3.00	0.34	0.03
TsOYE	EtOH	5			0.44	0.07
TsOYE	EtOH	10	85.67	1.53	0.50	0.04
TsOYE	EtOH	15	78.67	2.08	0.57	0.03
TsOYE	EtOH	20	67.67	2.08	0.62	0.01
TsOYE	EtOH	25	63.67	2.08	0.69	0.03
TsOYE	EtOH	30	56.33	3.21	0.70	0.01
TsOYE	MeOH	0	92.00	3.00	1.21	0.04
TsOYE	MeOH	5			1.11	0.08
TsOYE	MeOH	10	88.00	1.00	1.16	0.13
TsOYE	MeOH	15	84.00	0.00	1.13	0.11
TsOYE	MeOH	20	81.33	1.53	1.09	0.11
TsOYE	MeOH	25	75.00	1.00	0.84	0.06
TsOYE	MeOH	30	68.67	3.51	0.80	0.02
TsOYE	iprop	0	92.00	3.00	1.26	0.15
TsOYE	iprop	5	83.00	1.73	1.78	0.25
TsOYE	iprop	10	77.00	1.73	1.77	0.02
TsOYE	iprop	15	71.67	0.58	1.87	0.28
TsOYE	iprop	20	64.00	1.73	2.38	0.30
TsOYE	iprop	25	57.33	0.58	1.89	0.18
TsOYE	iprop	30	52.67	0.58	1.98	0.10
TsOYE	nprop	0	92.00	3.00	1.32	0.15
TsOYE	nprop	5	86.67	0.58	1.56	0.05
TsOYE	nprop	10	72.00	1.73	1.65	0.07
TsOYE	nprop	15	57.67	2.31	1.71	0.10
TsOYE	nprop	20	49.00	1.00	1.88	0.15
TsOYE	nprop	25	46.00	1.00	1.53	0.15
TsOYE	nprop	30	44.67	1.15	1.14	0.07
XenA	DMSO	0	49.00	0.00	0.21	0.02
XenA	DMSO	5	48.00	0.00	0.16	0.01
XenA	DMSO	10	47.00	0.00	0.13	0.00
XenA	DMSO	15	47.00	0.00	0.09	0.00
XenA	DMSO	20	46.33	0.58	0.05	0.00
XenA	DMSO	25	46.00	0.00	0.02	0.00
XenA	DMSO	30	46.00	0.00	0.00	0.03
XenA	EtOH	0	49.00	0.00	0.19	0.01
XenA	EtOH	5	45.67	0.58	0.20	0.02
XenA	EtOH	10	41.00	0.00	0.17	0.01
XenA	EtOH	15	38.33	1.15	0.16	0.02

XenA	EtOH	20	34.67	0.58	0.08	0.04
XenA	EtOH	25	34.00	1.00	0.06	0.01
XenA	EtOH	30	30.33	1.15	0.00	0.01
XenA	MeOH	0	49.00	0.00	0.12	0.03
XenA	MeOH	5	47.33	0.58	0.16	0.02
XenA	MeOH	10	44.33	0.58	0.20	0.00
XenA	MeOH	15	42.33	0.58	0.20	0.00
XenA	MeOH	20	38.67	0.58	0.17	0.00
XenA	MeOH	25	35.67	0.58	0.06	0.00
XenA	MeOH	30	33.00	1.00	0.00	0.01
XenA	iprop	0	49.00	0.00	0.19	0.02
XenA	iprop	5	44.00	1.00	0.18	0.05
XenA	iprop	10	38.00	1.00	0.16	0.02
XenA	iprop	15	34.33	2.08	0.13	0.01
XenA	iprop	20	30.33	2.08	0.02	0.03
XenA	iprop	25	27.67	1.15	0.00	0.06
XenA	iprop	30	23.33	3.51	0.00	0.05
XenA	nprop	0	49.00	0.00	0.19	0.02
XenA	nprop	5	41.67	1.53	0.11	0.01
XenA	nprop	10	32.33	1.53	0.06	0.00
XenA	nprop	15	29.00	1.00	0.02	0.00
XenA	nprop	20	28.00	1.00	0.00	0.02
XenA	nprop	25	27.33	0.58	0.00	0.01
XenA	nprop	30	24.67	4.04	0.00	0.03
XenB	DMSO	0	45.00	0.00	1.52	0.10
XenB	DMSO	5	42.33	0.58	1.15	0.06
XenB	DMSO	10	41.00	0.00	0.86	0.10
XenB	DMSO	15	40.00	0.00	0.74	0.01
XenB	DMSO	20	38.33	0.58	0.57	0.01
XenB	DMSO	25	38.00	0.00	0.41	0.01
XenB	DMSO	30	37.00	0.00	0.21	0.01
XenB	EtOH	0	45.00	0.00	1.65	0.37
XenB	EtOH	5	42.00	1.00	1.44	0.08
XenB	EtOH	10	38.33	0.58	1.46	0.01
XenB	EtOH	15	35.33	0.58	1.40	0.03
XenB	EtOH	20	32.33	1.53	1.34	0.10
XenB	EtOH	25	31.67	1.15	1.19	0.04
XenB	EtOH	30	31.00	0.00	0.81	0.03
XenB	MeOH	0	45.00	0.00	1.42	0.03
XenB	MeOH	5	43.00	0.00	1.42	0.03
XenB	MeOH	10	39.67	2.31	1.37	0.08
XenB	MeOH	15	37.33	1.15	1.29	0.01
XenB	MeOH	20	36.00	0.00	1.11	0.05
XenB	MeOH	25	34.67	0.58	0.76	0.04
XenB	MeOH	30	31.67	0.58	0.62	0.28
XenB	iprop	0	45.00	0.00	1.38	0.04
XenB	iprop	5	40.00	1.00	1.36	0.07

XenB	iprop	10	36.33	1.53	1.37	0.04
XenB	iprop	15	36.33	0.58	1.26	0.05
XenB	iprop	20	32.67	0.58	1.02	0.04
XenB	iprop	25	31.00	0.00	0.67	0.08
XenB	iprop	30	29.33	0.58	0.33	0.08
XenB	nprop	0	45.00	0.00	1.33	0.08
XenB	nprop	5	38.67	1.15	1.39	0.02
XenB	nprop	10	33.33	0.58	1.25	0.04
XenB	nprop	15	30.00	1.00	0.92	0.04
XenB	nprop	20	28.67	0.58	0.54	0.11
XenB	nprop	25	28.00	1.00	0.19	0.03
XenB	nprop	30	28.00	0.00	0.00	0.02
YqiG	DMSO	0	54.00	0.00	4.09	0.12
YqiG	DMSO	5	51.67	0.58	4.40	0.11
YqiG	DMSO	10	51.00	0.00	4.34	0.17
YqiG	DMSO	15	50.00	0.00	4.41	0.29
YqiG	DMSO	20	50.00	0.00	4.20	0.38
YqiG	DMSO	25	49.67	0.58	4.35	0.44
YqiG	DMSO	30	49.67	0.58	4.20	0.46
YqiG	EtOH	0	54.00	0.00	5.73	0.43
YqiG	EtOH	5	51.00	1.00	5.90	0.14
YqiG	EtOH	10	45.33	1.15	6.22	0.39
YqiG	EtOH	15	42.00	1.00	6.67	0.56
YqiG	EtOH	20	38.67	0.58	6.21	0.70
YqiG	EtOH	25	38.33	0.58	6.62	0.84
YqiG	EtOH	30	36.33	0.58	5.96	0.43
YqiG	MeOH	0	54.00	0.00	5.71	0.54
YqiG	MeOH	5	52.33	0.58	6.14	0.51
YqiG	MeOH	10	49.67	0.58	5.62	0.49
YqiG	MeOH	15	43.67	2.52	5.36	0.24
YqiG	MeOH	20	44.33	0.58	5.05	0.21
YqiG	MeOH	25	42.33	1.15	5.09	0.59
YqiG	MeOH	30	40.00	3.61	4.27	0.27
YqiG	iprop	0	54.00	0.00	5.75	0.28
YqiG	iprop	5	46.00	0.00	6.02	0.29
YqiG	iprop	10	39.33	1.15	6.11	0.24
YqiG	iprop	15	39.33	4.51	6.21	0.37
YqiG	iprop	20	33.67	0.58	6.17	0.29
YqiG	iprop	25	32.67	1.15	5.81	0.49
YqiG	iprop	30	33.67	0.58	5.26	0.24
YqiG	nprop	0	54.00	0.00	5.25	0.46
YqiG	nprop	5	43.67	2.08	5.20	0.41
YqiG	nprop	10	33.00	1.00	4.82	0.17
YqiG	nprop	15	31.33	0.58	4.55	0.20
YqiG	nprop	20	30.33	0.58	4.07	0.42
YqiG	nprop	25	29.33	0.58	3.69	0.30
YqiG	nprop	30	30.33	0.58	2.94	0.14

YqjM	DMSO	0	51.00	0.00	0.52	0.07
YqjM	DMSO	5	49.33	0.58	0.40	0.01
YqjM	DMSO	10	48.00	0.00	0.33	0.01
YqjM	DMSO	15	46.67	0.58	0.25	0.01
YqjM	DMSO	20	46.00	0.00	0.17	0.02
YqjM	DMSO	25	45.00	0.00	0.10	0.01
YqjM	DMSO	30	44.00	0.00	0.05	0.05
YqjM	EtOH	0	51.00	0.00	0.46	0.03
YqjM	EtOH	5	45.00	1.73	0.33	0.01
YqjM	EtOH	10	42.33	0.58	0.21	0.03
YqjM	EtOH	15	38.33	0.58	0.07	0.02
YqjM	EtOH	20	35.00	1.73	0.03	0.01
YqjM	EtOH	25	33.00	1.00	0.03	0.01
YqjM	EtOH	30	31.00	0.00	0.02	0.01
YqjM	MeOH	0	51.00	0.00	0.51	0.12
YqjM	MeOH	5	48.33	0.58	0.39	0.01
YqjM	MeOH	10	43.33	1.53	0.30	0.01
YqjM	MeOH	15	40.00	1.73	0.13	0.02
YqjM	MeOH	20	39.33	1.15	0.12	0.09
YqjM	MeOH	25	36.67	0.58	0.07	0.05
YqjM	MeOH	30	33.00	1.00	0.05	0.03
YqjM	iprop	0	50.67	0.58	0.49	0.07
YqjM	iprop	5	40.00	1.00	0.34	0.03
YqjM	iprop	10	36.33	0.58	0.21	0.01
YqjM	iprop	15	34.00	1.00	0.16	0.05
YqjM	iprop	20	31.33	0.58	0.11	0.07
YqjM	iprop	25	27.33	0.58	0.14	0.03
YqjM	iprop	30	24.33	3.79	0.06	0.09
YqjM	nprop	0	51.00	0.00	0.50	0.12
YqjM	nprop	5	43.33	1.15	0.25	0.02
YqjM	nprop	10	35.33	0.58	0.17	0.01
YqjM	nprop	15	29.67	0.58	0.12	0.01
YqjM	nprop	20	28.33	0.58	0.05	0.03
YqjM	nprop	25	27.67	0.58	0.00	0.03
YqjM	nprop	30	26.67	0.58	0.00	0.08

Supplementary Table 6 Raw activity data of reaction temperature screening

Enzyme	Solvent	Concentration	Temperature[°C]	Activity_mean [U mg^{-1}]	Activity_std [U mg^{-1}]
ChrOYE1	EtOH	0	25	0.48	0.23
ChrOYE1	EtOH	5	25	0.55	0.30
ChrOYE1	EtOH	10	25	0.58	0.28
ChrOYE1	EtOH	15	25	0.51	0.28
ChrOYE1	EtOH	20	25	0.45	0.31
ChrOYE1	EtOH	25	25	0.28	0.21
ChrOYE1	EtOH	30	25	0.16	0.11
ChrOYE1	EtOH	35	25	0.14	0.09
ChrOYE1	EtOH	40	25	0.11	0.11
ChrOYE1	EtOH	45	25	0.04	0.04
ChrOYE1	EtOH	no enzyme	25	0.00	0.01
ChrOYE1	EtOH	no substrate	25	0.10	0.10
ChrOYE1	EtOH	0	30	0.66	0.15
ChrOYE1	EtOH	5	30	0.81	0.05
ChrOYE1	EtOH	10	30	0.95	0.07
ChrOYE1	EtOH	15	30	0.92	0.04
ChrOYE1	EtOH	20	30	0.72	0.03
ChrOYE1	EtOH	25	30	0.41	0.02
ChrOYE1	EtOH	30	30	0.37	0.12
ChrOYE1	EtOH	35	30	0.13	0.05
ChrOYE1	EtOH	40	30	0.11	0.10
ChrOYE1	EtOH	45	30	0.03	0.02
ChrOYE1	EtOH	no enzyme	30	0.00	0.01
ChrOYE1	EtOH	no substrate	30	0.02	0.02
ChrOYE1	EtOH	0	35	0.57	0.05
ChrOYE1	EtOH	5	35	0.73	0.05
ChrOYE1	EtOH	10	35	0.74	0.04
ChrOYE1	EtOH	15	35	0.60	0.10
ChrOYE1	EtOH	20	35	0.32	0.07
ChrOYE1	EtOH	25	35	0.11	0.03
ChrOYE1	EtOH	30	35	0.04	0.01
ChrOYE1	EtOH	35	35	0.05	0.01
ChrOYE1	EtOH	40	35	0.04	0.01
ChrOYE1	EtOH	45	35	0.03	0.01
ChrOYE1	EtOH	no enzyme	35	0.00	0.03
ChrOYE1	EtOH	no substrate	35	0.05	0.01
ChrOYE1	EtOH	0	40	0.35	0.07
ChrOYE1	EtOH	5	40	0.39	0.04
ChrOYE1	EtOH	10	40	0.28	0.08
ChrOYE1	EtOH	15	40	0.17	0.03
ChrOYE1	EtOH	20	40	0.08	0.04
ChrOYE1	EtOH	25	40	0.07	0.02

ChrOYE1	EtOH	30	40	0.04	0.02
ChrOYE1	EtOH	35	40	0.05	0.01
ChrOYE1	EtOH	40	40	0.05	0.02
ChrOYE1	EtOH	45	40	0.02	0.02
ChrOYE1	EtOH	no enzyme	40	0.00	0.01
ChrOYE1	EtOH	no substrate	40	0.09	0.04
ChrOYE1	EtOH	0	45	0.48	0.09
ChrOYE1	EtOH	5	45	0.53	0.10
ChrOYE1	EtOH	10	45	0.47	0.06
ChrOYE1	EtOH	15	45	0.20	0.08
ChrOYE1	EtOH	20	45	0.07	0.03
ChrOYE1	EtOH	25	45	0.05	0.02
ChrOYE1	EtOH	30	45	0.02	0.00
ChrOYE1	EtOH	35	45	0.04	0.01
ChrOYE1	EtOH	40	45	0.04	0.01
ChrOYE1	EtOH	45	45	0.01	0.01
ChrOYE1	EtOH	no enzyme	45	0.00	0.05
ChrOYE1	EtOH	no substrate	45	0.04	0.02
ChrOYE1	MeOH	0	25	0.56	0.11
ChrOYE1	MeOH	5	25	0.61	0.12
ChrOYE1	MeOH	10	25	0.61	0.01
ChrOYE1	MeOH	15	25	0.66	0.12
ChrOYE1	MeOH	20	25	0.60	0.04
ChrOYE1	MeOH	25	25	0.32	0.02
ChrOYE1	MeOH	30	25	0.13	0.03
ChrOYE1	MeOH	35	25	0.07	0.03
ChrOYE1	MeOH	40	25	0.08	0.01
ChrOYE1	MeOH	45	25	0.03	0.01
ChrOYE1	MeOH	no enzyme	25	0.00	0.02
ChrOYE1	MeOH	no substrate	25	0.07	0.03
ChrOYE1	MeOH	0	30	0.61	0.03
ChrOYE1	MeOH	5	30	0.62	0.03
ChrOYE1	MeOH	10	30	0.78	0.03
ChrOYE1	MeOH	15	30	0.77	0.00
ChrOYE1	MeOH	20	30	0.67	0.01
ChrOYE1	MeOH	25	30	0.45	0.02
ChrOYE1	MeOH	30	30	0.19	0.04
ChrOYE1	MeOH	35	30	0.05	0.01
ChrOYE1	MeOH	40	30	0.03	0.01
ChrOYE1	MeOH	45	30	0.04	0.03
ChrOYE1	MeOH	no enzyme	30	0.00	0.01
ChrOYE1	MeOH	no substrate	30	0.04	0.01
ChrOYE1	MeOH	0	35	0.53	0.02
ChrOYE1	MeOH	5	35	0.61	0.02
ChrOYE1	MeOH	10	35	0.63	0.06
ChrOYE1	MeOH	15	35	0.60	0.02
ChrOYE1	MeOH	20	35	0.43	0.04

ChrOYE1	MeOH	25	35	0.18	0.04
ChrOYE1	MeOH	30	35	0.05	0.01
ChrOYE1	MeOH	35	35	0.01	0.01
ChrOYE1	MeOH	40	35	0.02	0.01
ChrOYE1	MeOH	45	35	0.01	0.01
ChrOYE1	MeOH	no enzyme	35	0.00	0.03
ChrOYE1	MeOH	no substrate	35	0.05	0.01
ChrOYE1	MeOH	0	40	0.29	0.09
ChrOYE1	MeOH	5	40	0.29	0.05
ChrOYE1	MeOH	10	40	0.29	0.06
ChrOYE1	MeOH	15	40	0.17	0.02
ChrOYE1	MeOH	20	40	0.09	0.02
ChrOYE1	MeOH	25	40	0.07	0.02
ChrOYE1	MeOH	30	40	0.03	0.02
ChrOYE1	MeOH	35	40	0.06	0.04
ChrOYE1	MeOH	40	40	0.06	0.05
ChrOYE1	MeOH	45	40	0.03	0.02
ChrOYE1	MeOH	no enzyme	40	0.00	0.01
ChrOYE1	MeOH	no substrate	40	0.07	0.02
ChrOYE1	MeOH	0	45	0.19	0.06
ChrOYE1	MeOH	5	45	0.36	0.11
ChrOYE1	MeOH	10	45	0.45	0.04
ChrOYE1	MeOH	15	45	0.23	0.09
ChrOYE1	MeOH	20	45	0.08	0.04
ChrOYE1	MeOH	25	45	0.06	0.02
ChrOYE1	MeOH	30	45	0.03	0.01
ChrOYE1	MeOH	35	45	0.02	0.00
ChrOYE1	MeOH	40	45	0.03	0.01
ChrOYE1	MeOH	45	45	0.04	0.03
ChrOYE1	MeOH	no enzyme	45	0.00	0.04
ChrOYE1	MeOH	no substrate	45	0.05	0.01
DrER	EtOH	0	25	0.72	0.46
DrER	EtOH	5	25	0.67	0.20
DrER	EtOH	10	25	1.05	0.05
DrER	EtOH	15	25	0.98	0.11
DrER	EtOH	20	25	0.68	0.02
DrER	EtOH	25	25	0.37	0.16
DrER	EtOH	30	25	0.18	0.05
DrER	EtOH	35	25	0.14	0.03
DrER	EtOH	40	25	0.04	0.11
DrER	EtOH	45	25	0.11	0.08
DrER	EtOH	no enzyme	25	0.00	0.22
DrER	EtOH	no substrate	25	0.12	0.08
DrER	EtOH	0	30	1.06	0.08
DrER	EtOH	5	30	1.10	0.09
DrER	EtOH	10	30	1.14	0.03
DrER	EtOH	15	30	1.00	0.04

DrER	EtOH	20	30	0.44	0.06
DrER	EtOH	25	30	0.10	0.03
DrER	EtOH	30	30	0.06	0.02
DrER	EtOH	35	30	0.00	0.01
DrER	EtOH	40	30	0.02	0.05
DrER	EtOH	45	30	0.10	0.07
DrER	EtOH	no enzyme	30	0.06	0.04
DrER	EtOH	no substrate	30	0.04	0.03
DrER	EtOH	0	35	1.44	0.25
DrER	EtOH	5	35	1.13	0.15
DrER	EtOH	10	35	1.23	0.11
DrER	EtOH	15	35	0.57	0.05
DrER	EtOH	20	35	0.15	0.02
DrER	EtOH	25	35	0.02	0.02
DrER	EtOH	30	35	0.05	0.02
DrER	EtOH	35	35	0.03	0.02
DrER	EtOH	40	35	0.03	0.04
DrER	EtOH	45	35	0.01	0.04
DrER	EtOH	no enzyme	35	0.02	0.02
DrER	EtOH	no substrate	35	0.07	0.08
DrER	EtOH	0	40	0.61	0.04
DrER	EtOH	5	40	0.60	0.21
DrER	EtOH	10	40	0.21	0.02
DrER	EtOH	15	40	0.04	0.01
DrER	EtOH	20	40	0.05	0.01
DrER	EtOH	25	40	0.04	0.02
DrER	EtOH	30	40	0.04	0.02
DrER	EtOH	35	40	0.03	0.01
DrER	EtOH	40	40	0.11	0.01
DrER	EtOH	45	40	0.00	0.07
DrER	EtOH	no enzyme	40	0.03	0.04
DrER	EtOH	no substrate	40	0.01	0.01
DrER	EtOH	0	45	0.29	0.08
DrER	EtOH	5	45	0.20	0.03
DrER	EtOH	10	45	0.02	0.02
DrER	EtOH	15	45	0.04	0.03
DrER	EtOH	20	45	0.08	0.06
DrER	EtOH	25	45	0.04	0.03
DrER	EtOH	30	45	0.04	0.01
DrER	EtOH	35	45	0.00	0.19
DrER	EtOH	40	45	0.06	0.00
DrER	EtOH	45	45	0.02	0.04
DrER	EtOH	no enzyme	45	0.00	0.00
DrER	EtOH	no substrate	45	0.00	0.00
DrER	MeOH	0	25	0.95	0.12
DrER	MeOH	5	25	0.89	0.12
DrER	MeOH	10	25	1.10	0.15

DrER	MeOH	15	25	1.01	0.06
DrER	MeOH	20	25	0.92	0.13
DrER	MeOH	25	25	0.60	0.12
DrER	MeOH	30	25	0.19	0.01
DrER	MeOH	35	25	0.11	0.02
DrER	MeOH	40	25	0.12	0.04
DrER	MeOH	45	25	0.15	0.04
DrER	MeOH	no enzyme	25	0.00	0.14
DrER	MeOH	no substrate	25	0.15	0.22
DrER	MeOH	0	30	0.92	0.07
DrER	MeOH	5	30	0.95	0.07
DrER	MeOH	10	30	1.11	0.07
DrER	MeOH	15	30	1.03	0.05
DrER	MeOH	20	30	0.83	0.10
DrER	MeOH	25	30	0.39	0.07
DrER	MeOH	30	30	0.09	0.04
DrER	MeOH	35	30	0.00	0.01
DrER	MeOH	40	30	0.03	0.06
DrER	MeOH	45	30	0.03	0.04
DrER	MeOH	no enzyme	30	0.02	0.03
DrER	MeOH	no substrate	30	0.05	0.02
DrER	MeOH	0	35	0.97	0.14
DrER	MeOH	5	35	0.99	0.04
DrER	MeOH	10	35	0.98	0.04
DrER	MeOH	15	35	0.89	0.02
DrER	MeOH	20	35	0.32	0.05
DrER	MeOH	25	35	0.07	0.04
DrER	MeOH	30	35	0.05	0.00
DrER	MeOH	35	35	0.02	0.02
DrER	MeOH	40	35	0.07	0.01
DrER	MeOH	45	35	0.03	0.02
DrER	MeOH	no enzyme	35	0.05	0.03
DrER	MeOH	no substrate	35	0.01	0.01
DrER	MeOH	0	40	0.67	0.01
DrER	MeOH	5	40	0.68	0.16
DrER	MeOH	10	40	0.41	0.03
DrER	MeOH	15	40	0.20	0.03
DrER	MeOH	20	40	0.05	0.03
DrER	MeOH	25	40	0.02	0.01
DrER	MeOH	30	40	0.03	0.00
DrER	MeOH	35	40	0.03	0.01
DrER	MeOH	40	40	0.05	0.02
DrER	MeOH	45	40	0.02	0.01
DrER	MeOH	no enzyme	40	0.02	0.01
DrER	MeOH	no substrate	40	0.05	0.03
DrER	MeOH	0	45	0.35	0.02
DrER	MeOH	5	45	0.28	0.05

DrER	MeOH	10	45	0.12	0.04
DrER	MeOH	15	45	0.01	0.03
DrER	MeOH	20	45	0.03	0.02
DrER	MeOH	25	45	0.03	0.02
DrER	MeOH	30	45	0.03	0.01
DrER	MeOH	35	45	0.04	0.01
DrER	MeOH	40	45	0.06	0.04
DrER	MeOH	45	45	0.03	0.01
DrER	MeOH	no enzyme	45	0.00	0.00
DrER	MeOH	no substrate	45	0.00	0.00
NCR	EtOH	0	25	0.45	0.06
NCR	EtOH	5	25	0.44	0.01
NCR	EtOH	10	25	0.37	0.03
NCR	EtOH	15	25	0.29	0.00
NCR	EtOH	20	25	0.20	0.01
NCR	EtOH	25	25	0.11	0.01
NCR	EtOH	30	25	0.09	0.07
NCR	EtOH	35	25	0.04	0.01
NCR	EtOH	40	25	0.02	0.02
NCR	EtOH	45	25	0.00	0.06
NCR	EtOH	no enzyme	25	0.02	0.01
NCR	EtOH	no substrate	25	0.02	0.01
NCR	EtOH	0	30	0.58	0.07
NCR	EtOH	5	30	0.53	0.01
NCR	EtOH	10	30	0.41	0.02
NCR	EtOH	15	30	0.31	0.01
NCR	EtOH	20	30	0.21	0.01
NCR	EtOH	25	30	0.12	0.01
NCR	EtOH	30	30	0.05	0.02
NCR	EtOH	35	30	0.03	0.01
NCR	EtOH	40	30	0.00	0.01
NCR	EtOH	45	30	0.00	0.01
NCR	EtOH	no enzyme	30	0.03	0.00
NCR	EtOH	no substrate	30	0.07	0.04
NCR	EtOH	0	35	0.65	0.02
NCR	EtOH	5	35	0.57	0.02
NCR	EtOH	10	35	0.48	0.03
NCR	EtOH	15	35	0.30	0.05
NCR	EtOH	20	35	0.12	0.04
NCR	EtOH	25	35	0.06	0.02
NCR	EtOH	30	35	0.02	0.01
NCR	EtOH	35	35	0.03	0.01
NCR	EtOH	40	35	0.05	0.02
NCR	EtOH	45	35	0.09	0.04
NCR	EtOH	no enzyme	35	0.05	0.02
NCR	EtOH	no substrate	35	0.09	0.03
NCR	EtOH	0	40	0.41	0.05

NCR	EtOH	5	40	0.33	0.04
NCR	EtOH	10	40	0.19	0.05
NCR	EtOH	15	40	0.07	0.04
NCR	EtOH	20	40	0.02	0.01
NCR	EtOH	25	40	0.02	0.02
NCR	EtOH	30	40	0.02	0.01
NCR	EtOH	35	40	0.03	0.01
NCR	EtOH	40	40	0.02	0.01
NCR	EtOH	45	40	0.05	0.01
NCR	EtOH	no enzyme	40	0.08	0.01
NCR	EtOH	no substrate	40	0.03	0.03
NCR	EtOH	0	45	0.12	0.04
NCR	EtOH	5	45	0.04	0.03
NCR	EtOH	10	45	0.14	0.10
NCR	EtOH	15	45	0.03	0.03
NCR	EtOH	20	45	0.01	0.01
NCR	EtOH	25	45	0.06	0.01
NCR	EtOH	30	45	0.02	0.02
NCR	EtOH	35	45	0.05	0.02
NCR	EtOH	40	45	0.08	0.05
NCR	EtOH	45	45	0.03	0.02
NCR	EtOH	no enzyme	45	0.10	0.00
NCR	EtOH	no substrate	45	0.02	0.05
NCR	MeOH	0	25	0.46	0.03
NCR	MeOH	5	25	0.47	0.02
NCR	MeOH	10	25	0.45	0.02
NCR	MeOH	15	25	0.38	0.02
NCR	MeOH	20	25	0.32	0.02
NCR	MeOH	25	25	0.22	0.01
NCR	MeOH	30	25	0.16	0.02
NCR	MeOH	35	25	0.07	0.01
NCR	MeOH	40	25	0.04	0.01
NCR	MeOH	45	25	0.01	0.01
NCR	MeOH	no enzyme	25	0.03	0.01
NCR	MeOH	no substrate	25	0.04	0.01
NCR	MeOH	0	30	0.62	0.17
NCR	MeOH	5	30	0.58	0.08
NCR	MeOH	10	30	0.49	0.05
NCR	MeOH	15	30	0.41	0.04
NCR	MeOH	20	30	0.31	0.05
NCR	MeOH	25	30	0.24	0.04
NCR	MeOH	30	30	0.13	0.05
NCR	MeOH	35	30	0.04	0.04
NCR	MeOH	40	30	0.03	0.01
NCR	MeOH	45	30	0.05	0.01
NCR	MeOH	no enzyme	30	0.04	0.01
NCR	MeOH	no substrate	30	0.01	0.06

NCR	MeOH	0	35	0.61	0.04
NCR	MeOH	5	35	0.59	0.01
NCR	MeOH	10	35	0.48	0.06
NCR	MeOH	15	35	0.41	0.03
NCR	MeOH	20	35	0.28	0.02
NCR	MeOH	25	35	0.15	0.03
NCR	MeOH	30	35	0.02	0.04
NCR	MeOH	35	35	0.01	0.01
NCR	MeOH	40	35	0.00	0.02
NCR	MeOH	45	35	0.02	0.00
NCR	MeOH	no enzyme	35	0.04	0.01
NCR	MeOH	no substrate	35	0.02	0.04
NCR	MeOH	0	40	0.43	0.14
NCR	MeOH	5	40	0.33	0.09
NCR	MeOH	10	40	0.23	0.04
NCR	MeOH	15	40	0.14	0.03
NCR	MeOH	20	40	0.03	0.04
NCR	MeOH	25	40	0.00	0.05
NCR	MeOH	30	40	0.01	0.03
NCR	MeOH	35	40	0.01	0.01
NCR	MeOH	40	40	0.01	0.01
NCR	MeOH	45	40	0.03	0.02
NCR	MeOH	no enzyme	40	0.07	0.01
NCR	MeOH	no substrate	40	0.05	0.04
NCR	MeOH	0	45	0.04	0.02
NCR	MeOH	5	45	0.04	0.05
NCR	MeOH	10	45	0.00	0.16
NCR	MeOH	15	45	0.04	0.03
NCR	MeOH	20	45	0.00	0.09
NCR	MeOH	25	45	0.00	0.04
NCR	MeOH	30	45	0.01	0.05
NCR	MeOH	35	45	0.04	0.00
NCR	MeOH	40	45	0.04	0.00
NCR	MeOH	45	45	0.03	0.02
NCR	MeOH	no enzyme	45	0.11	0.01
NCR	MeOH	no substrate	45	0.00	0.02
NerA	EtOH	0	25	0.02	0.00
NerA	EtOH	5	25	0.02	0.00
NerA	EtOH	10	25	0.02	0.00
NerA	EtOH	15	25	0.01	0.00
NerA	EtOH	20	25	0.01	0.00
NerA	EtOH	25	25	0.00	0.00
NerA	EtOH	30	25	0.00	0.00
NerA	EtOH	35	25	0.00	0.00
NerA	EtOH	40	25	0.00	0.00
NerA	EtOH	45	25	0.00	0.00
NerA	EtOH	no enzyme	25	0.00	0.00

NerA	EtOH	no substrate	25	0.02	0.00
NerA	EtOH	0	30	0.02	0.00
NerA	EtOH	5	30	0.02	0.00
NerA	EtOH	10	30	0.02	0.00
NerA	EtOH	15	30	0.01	0.00
NerA	EtOH	20	30	0.00	0.00
NerA	EtOH	25	30	0.00	0.00
NerA	EtOH	30	30	0.00	0.00
NerA	EtOH	35	30	0.00	0.00
NerA	EtOH	40	30	0.00	0.00
NerA	EtOH	45	30	0.00	0.00
NerA	EtOH	no enzyme	30	0.00	0.00
NerA	EtOH	no substrate	30	0.02	0.00
NerA	MeOH	0	25	0.02	0.00
NerA	MeOH	5	25	0.02	0.00
NerA	MeOH	10	25	0.02	0.00
NerA	MeOH	15	25	0.02	0.00
NerA	MeOH	20	25	0.02	0.00
NerA	MeOH	25	25	0.01	0.00
NerA	MeOH	30	25	0.00	0.00
NerA	MeOH	35	25	0.00	0.00
NerA	MeOH	40	25	0.00	0.00
NerA	MeOH	45	25	0.00	0.00
NerA	MeOH	no enzyme	25	0.00	0.00
NerA	MeOH	no substrate	25	0.02	0.00
NerA	MeOH	0	30	0.02	0.00
NerA	MeOH	5	30	0.02	0.00
NerA	MeOH	10	30	0.02	0.00
NerA	MeOH	15	30	0.02	0.00
NerA	MeOH	20	30	0.02	0.00
NerA	MeOH	25	30	0.00	0.00
NerA	MeOH	30	30	0.00	0.00
NerA	MeOH	35	30	0.00	0.00
NerA	MeOH	40	30	0.00	0.00
NerA	MeOH	45	30	0.00	0.00
NerA	MeOH	no enzyme	30	0.00	0.00
NerA	MeOH	no substrate	30	0.02	0.00
OYE1	EtOH	0	25	0.20	0.02
OYE1	EtOH	5	25	0.28	0.05
OYE1	EtOH	10	25	0.27	0.02
OYE1	EtOH	15	25	0.32	0.02
OYE1	EtOH	20	25	0.36	0.02
OYE1	EtOH	25	25	0.27	0.03
OYE1	EtOH	30	25	0.15	0.05
OYE1	EtOH	35	25	0.07	0.04
OYE1	EtOH	40	25	0.02	0.02
OYE1	EtOH	45	25	0.01	0.02

OYE1	EtOH	no enzyme	25	0.02	0.03
OYE1	EtOH	no substrate	25	0.02	0.04
OYE1	EtOH	0	30	0.23	0.03
OYE1	EtOH	5	30	0.32	0.02
OYE1	EtOH	10	30	0.37	0.02
OYE1	EtOH	15	30	0.36	0.01
OYE1	EtOH	20	30	0.35	0.02
OYE1	EtOH	25	30	0.21	0.02
OYE1	EtOH	30	30	0.15	0.06
OYE1	EtOH	35	30	0.03	0.03
OYE1	EtOH	40	30	0.02	0.01
OYE1	EtOH	45	30	0.04	0.04
OYE1	EtOH	no enzyme	30	0.01	0.01
OYE1	EtOH	no substrate	30	0.07	0.02
OYE1	EtOH	0	35	0.36	0.02
OYE1	EtOH	5	35	0.47	0.02
OYE1	EtOH	10	35	0.52	0.03
OYE1	EtOH	15	35	0.52	0.05
OYE1	EtOH	20	35	0.32	0.09
OYE1	EtOH	25	35	0.03	0.02
OYE1	EtOH	30	35	0.02	0.02
OYE1	EtOH	35	35	0.01	0.02
OYE1	EtOH	40	35	0.01	0.00
OYE1	EtOH	45	35	0.02	0.00
OYE1	EtOH	no enzyme	35	0.02	0.00
OYE1	EtOH	no substrate	35	0.07	0.02
OYE1	EtOH	0	40	0.33	0.18
OYE1	EtOH	5	40	0.34	0.08
OYE1	EtOH	10	40	0.21	0.12
OYE1	EtOH	15	40	0.09	0.05
OYE1	EtOH	20	40	0.02	0.00
OYE1	EtOH	25	40	0.01	0.01
OYE1	EtOH	30	40	0.01	0.01
OYE1	EtOH	35	40	0.01	0.01
OYE1	EtOH	40	40	0.01	0.01
OYE1	EtOH	45	40	0.01	0.02
OYE1	EtOH	no enzyme	40	0.04	0.00
OYE1	EtOH	no substrate	40	0.09	0.02
OYE1	EtOH	0	45	0.10	0.12
OYE1	EtOH	5	45	0.17	0.10
OYE1	EtOH	10	45	0.12	0.08
OYE1	EtOH	15	45	0.05	0.00
OYE1	EtOH	20	45	0.02	0.03
OYE1	EtOH	25	45	0.01	0.01
OYE1	EtOH	30	45	0.02	0.01
OYE1	EtOH	35	45	0.03	0.00
OYE1	EtOH	40	45	0.01	0.01

OYE1	EtOH	45	45	0.02	0.01
OYE1	EtOH	no enzyme	45	0.04	0.00
OYE1	EtOH	no substrate	45	0.04	0.04
OYE1	MeOH	0	25	0.24	0.02
OYE1	MeOH	5	25	0.21	0.12
OYE1	MeOH	10	25	0.30	0.02
OYE1	MeOH	15	25	0.33	0.03
OYE1	MeOH	20	25	0.37	0.04
OYE1	MeOH	25	25	0.31	0.06
OYE1	MeOH	30	25	0.19	0.03
OYE1	MeOH	35	25	0.03	0.02
OYE1	MeOH	40	25	0.02	0.02
OYE1	MeOH	45	25	0.01	0.00
OYE1	MeOH	no enzyme	25	0.00	0.03
OYE1	MeOH	no substrate	25	0.02	0.02
OYE1	MeOH	0	30	0.21	0.00
OYE1	MeOH	5	30	0.29	0.00
OYE1	MeOH	10	30	0.36	0.01
OYE1	MeOH	15	30	0.40	0.03
OYE1	MeOH	20	30	0.43	0.01
OYE1	MeOH	25	30	0.30	0.01
OYE1	MeOH	30	30	0.14	0.02
OYE1	MeOH	35	30	0.04	0.03
OYE1	MeOH	40	30	0.03	0.01
OYE1	MeOH	45	30	0.03	0.00
OYE1	MeOH	no enzyme	30	0.01	0.01
OYE1	MeOH	no substrate	30	0.07	0.01
OYE1	MeOH	0	35	0.37	0.00
OYE1	MeOH	5	35	0.46	0.02
OYE1	MeOH	10	35	0.52	0.07
OYE1	MeOH	15	35	0.55	0.04
OYE1	MeOH	20	35	0.40	0.08
OYE1	MeOH	25	35	0.06	0.04
OYE1	MeOH	30	35	0.01	0.00
OYE1	MeOH	35	35	0.01	0.00
OYE1	MeOH	40	35	0.00	0.01
OYE1	MeOH	45	35	0.01	0.00
OYE1	MeOH	no enzyme	35	0.03	0.00
OYE1	MeOH	no substrate	35	0.08	0.01
OYE1	MeOH	0	40	0.23	0.06
OYE1	MeOH	5	40	0.25	0.07
OYE1	MeOH	10	40	0.17	0.07
OYE1	MeOH	15	40	0.09	0.07
OYE1	MeOH	20	40	0.02	0.04
OYE1	MeOH	25	40	0.02	0.01
OYE1	MeOH	30	40	0.01	0.01
OYE1	MeOH	35	40	0.00	0.00

OYE1	MeOH	40	40	0.01	0.01
OYE1	MeOH	45	40	0.01	0.01
OYE1	MeOH	no enzyme	40	0.04	0.00
OYE1	MeOH	no substrate	40	0.03	0.02
OYE1	MeOH	0	45	0.07	0.05
OYE1	MeOH	5	45	0.12	0.10
OYE1	MeOH	10	45	0.08	0.07
OYE1	MeOH	15	45	0.04	0.04
OYE1	MeOH	20	45	0.01	0.01
OYE1	MeOH	25	45	0.02	0.01
OYE1	MeOH	30	45	0.00	0.01
OYE1	MeOH	35	45	0.00	0.00
OYE1	MeOH	40	45	0.01	0.01
OYE1	MeOH	45	45	0.00	0.01
OYE1	MeOH	no enzyme	45	0.05	0.00
OYE1	MeOH	no substrate	45	0.01	0.01
PpXenB	EtOH	0	25	0.91	0.08
PpXenB	EtOH	5	25	0.88	0.02
PpXenB	EtOH	10	25	1.22	0.06
PpXenB	EtOH	15	25	1.24	0.01
PpXenB	EtOH	20	25	1.17	0.04
PpXenB	EtOH	25	25	0.93	0.01
PpXenB	EtOH	30	25	0.51	0.07
PpXenB	EtOH	35	25	0.24	0.02
PpXenB	EtOH	40	25	0.10	0.06
PpXenB	EtOH	45	25	0.00	0.05
PpXenB	EtOH	no enzyme	25	0.04	0.01
PpXenB	EtOH	no substrate	25	0.00	0.05
PpXenB	EtOH	0	30	1.06	0.25
PpXenB	EtOH	5	30	0.82	0.14
PpXenB	EtOH	10	30	1.10	0.14
PpXenB	EtOH	15	30	1.21	0.16
PpXenB	EtOH	20	30	1.06	0.10
PpXenB	EtOH	25	30	0.77	0.03
PpXenB	EtOH	30	30	0.41	0.04
PpXenB	EtOH	35	30	0.11	0.03
PpXenB	EtOH	40	30	0.06	0.05
PpXenB	EtOH	45	30	0.01	0.04
PpXenB	EtOH	no enzyme	30	0.04	0.01
PpXenB	EtOH	no substrate	30	0.00	0.05
PpXenB	EtOH	0	35	0.76	0.11
PpXenB	EtOH	5	35	0.69	0.03
PpXenB	EtOH	10	35	0.97	0.05
PpXenB	EtOH	15	35	0.97	0.11
PpXenB	EtOH	20	35	0.52	0.11
PpXenB	EtOH	25	35	0.12	0.05
PpXenB	EtOH	30	35	0.03	0.01

PpXenB	EtOH	35	35	0.02	0.01
PpXenB	EtOH	40	35	0.01	0.01
PpXenB	EtOH	45	35	0.01	0.05
PpXenB	EtOH	no enzyme	35	0.06	0.00
PpXenB	EtOH	no substrate	35	0.02	0.06
PpXenB	EtOH	0	40	0.24	0.10
PpXenB	EtOH	5	40	0.34	0.04
PpXenB	EtOH	10	40	0.33	0.12
PpXenB	EtOH	15	40	0.13	0.07
PpXenB	EtOH	20	40	0.02	0.01
PpXenB	EtOH	25	40	0.04	0.02
PpXenB	EtOH	30	40	0.04	0.03
PpXenB	EtOH	35	40	0.02	0.00
PpXenB	EtOH	40	40	0.02	0.02
PpXenB	EtOH	45	40	0.01	0.01
PpXenB	EtOH	no enzyme	40	0.06	0.04
PpXenB	EtOH	no substrate	40	0.00	0.05
PpXenB	EtOH	0	45	0.16	0.09
PpXenB	EtOH	5	45	0.16	0.04
PpXenB	EtOH	10	45	0.13	0.04
PpXenB	EtOH	15	45	0.08	0.03
PpXenB	EtOH	20	45	0.04	0.02
PpXenB	EtOH	25	45	0.04	0.02
PpXenB	EtOH	30	45	0.04	0.04
PpXenB	EtOH	35	45	0.03	0.01
PpXenB	EtOH	40	45	0.03	0.01
PpXenB	EtOH	45	45	0.03	0.02
PpXenB	EtOH	no enzyme	45	0.11	0.00
PpXenB	EtOH	no substrate	45	0.00	0.02
PpXenB	MeOH	0	25	1.00	0.23
PpXenB	MeOH	5	25	0.75	0.04
PpXenB	MeOH	10	25	0.75	0.07
PpXenB	MeOH	15	25	0.96	0.04
PpXenB	MeOH	20	25	0.96	0.03
PpXenB	MeOH	25	25	0.84	0.03
PpXenB	MeOH	30	25	0.49	0.03
PpXenB	MeOH	35	25	0.18	0.02
PpXenB	MeOH	40	25	0.05	0.02
PpXenB	MeOH	45	25	0.01	0.02
PpXenB	MeOH	no enzyme	25	0.02	0.00
PpXenB	MeOH	no substrate	25	0.00	0.06
PpXenB	MeOH	0	30	1.16	0.21
PpXenB	MeOH	5	30	0.94	0.15
PpXenB	MeOH	10	30	0.87	0.09
PpXenB	MeOH	15	30	0.95	0.09
PpXenB	MeOH	20	30	0.99	0.04
PpXenB	MeOH	25	30	0.77	0.05

PpXenB	MeOH	30	30	0.41	0.01
PpXenB	MeOH	35	30	0.09	0.01
PpXenB	MeOH	40	30	0.02	0.01
PpXenB	MeOH	45	30	0.04	0.02
PpXenB	MeOH	no enzyme	30	0.04	0.01
PpXenB	MeOH	no substrate	30	0.00	0.04
PpXenB	MeOH	0	35	0.48	0.33
PpXenB	MeOH	5	35	0.40	0.14
PpXenB	MeOH	10	35	0.54	0.09
PpXenB	MeOH	15	35	0.77	0.04
PpXenB	MeOH	20	35	0.54	0.07
PpXenB	MeOH	25	35	0.24	0.10
PpXenB	MeOH	30	35	0.05	0.02
PpXenB	MeOH	35	35	0.03	0.02
PpXenB	MeOH	40	35	0.00	0.04
PpXenB	MeOH	45	35	0.03	0.03
PpXenB	MeOH	no enzyme	35	0.05	0.00
PpXenB	MeOH	no substrate	35	0.00	0.04
PpXenB	MeOH	0	40	0.18	0.10
PpXenB	MeOH	5	40	0.29	0.07
PpXenB	MeOH	10	40	0.30	0.20
PpXenB	MeOH	15	40	0.19	0.08
PpXenB	MeOH	20	40	0.09	0.00
PpXenB	MeOH	25	40	0.04	0.03
PpXenB	MeOH	30	40	0.04	0.04
PpXenB	MeOH	35	40	0.02	0.02
PpXenB	MeOH	40	40	0.02	0.01
PpXenB	MeOH	45	40	0.00	0.02
PpXenB	MeOH	no enzyme	40	0.06	0.00
PpXenB	MeOH	no substrate	40	0.00	0.04
PpXenB	MeOH	0	45	0.21	0.03
PpXenB	MeOH	5	45	0.14	0.01
PpXenB	MeOH	10	45	0.13	0.00
PpXenB	MeOH	15	45	0.08	0.02
PpXenB	MeOH	20	45	0.05	0.01
PpXenB	MeOH	25	45	0.04	0.01
PpXenB	MeOH	30	45	0.02	0.03
PpXenB	MeOH	35	45	0.01	0.00
PpXenB	MeOH	40	45	0.04	0.01
PpXenB	MeOH	45	45	0.00	0.01
PpXenB	MeOH	no enzyme	45	0.12	0.01
PpXenB	MeOH	no substrate	45	0.00	0.07
TsOYE	EtOH	0	25	1.20	0.03
TsOYE	EtOH	5	25	1.26	0.08
TsOYE	EtOH	10	25	1.38	0.08
TsOYE	EtOH	15	25	1.55	0.05
TsOYE	EtOH	20	25	1.45	0.01

TsOYE	EtOH	25	25	1.57	0.01
TsOYE	EtOH	30	25	1.28	0.05
TsOYE	EtOH	35	25	1.21	0.05
TsOYE	EtOH	40	25	0.75	0.04
TsOYE	EtOH	45	25	0.61	0.04
TsOYE	EtOH	no enzyme	25	0.05	0.02
TsOYE	EtOH	no substrate	25	0.12	0.00
TsOYE	EtOH	0	30	1.18	0.34
TsOYE	EtOH	5	30	1.48	0.04
TsOYE	EtOH	10	30	1.51	0.01
TsOYE	EtOH	15	30	1.57	0.03
TsOYE	EtOH	20	30	1.53	0.04
TsOYE	EtOH	25	30	1.41	0.08
TsOYE	EtOH	30	30	1.29	0.02
TsOYE	EtOH	35	30	1.13	0.04
TsOYE	EtOH	40	30	0.88	0.09
TsOYE	EtOH	45	30	0.50	0.07
TsOYE	EtOH	no enzyme	30	0.05	0.00
TsOYE	EtOH	no substrate	30	0.12	0.02
TsOYE	EtOH	0	35	1.58	0.13
TsOYE	EtOH	5	35	1.59	0.06
TsOYE	EtOH	10	35	1.67	0.06
TsOYE	EtOH	15	35	1.48	0.12
TsOYE	EtOH	20	35	1.49	0.12
TsOYE	EtOH	25	35	1.45	0.07
TsOYE	EtOH	30	35	1.29	0.03
TsOYE	EtOH	35	35	0.94	0.06
TsOYE	EtOH	40	35	0.43	0.04
TsOYE	EtOH	45	35	0.30	0.04
TsOYE	EtOH	no enzyme	35	0.07	0.02
TsOYE	EtOH	no substrate	35	0.25	0.06
TsOYE	EtOH	0	40	1.51	0.08
TsOYE	EtOH	5	40	1.70	0.06
TsOYE	EtOH	10	40	1.63	0.15
TsOYE	EtOH	15	40	1.73	0.09
TsOYE	EtOH	20	40	1.58	0.05
TsOYE	EtOH	25	40	1.48	0.03
TsOYE	EtOH	30	40	1.10	0.08
TsOYE	EtOH	35	40	0.69	0.07
TsOYE	EtOH	40	40	0.30	0.05
TsOYE	EtOH	45	40	0.17	0.04
TsOYE	EtOH	no enzyme	40	0.11	0.03
TsOYE	EtOH	no substrate	40	0.26	0.03
TsOYE	EtOH	0	45	1.41	0.11
TsOYE	EtOH	5	45	1.55	0.09
TsOYE	EtOH	10	45	1.73	0.05
TsOYE	EtOH	15	45	1.77	0.12

TsOYE	EtOH	20	45	1.60	0.04
TsOYE	EtOH	25	45	1.40	0.04
TsOYE	EtOH	30	45	1.02	0.09
TsOYE	EtOH	35	45	0.40	0.07
TsOYE	EtOH	40	45	0.16	0.05
TsOYE	EtOH	45	45	0.08	0.03
TsOYE	EtOH	no enzyme	45	0.07	0.01
TsOYE	EtOH	no substrate	45	0.31	0.02
TsOYE	MeOH	0	25	1.22	0.09
TsOYE	MeOH	5	25	1.14	0.07
TsOYE	MeOH	10	25	1.12	0.03
TsOYE	MeOH	15	25	1.09	0.06
TsOYE	MeOH	20	25	1.10	0.02
TsOYE	MeOH	25	25	0.99	0.02
TsOYE	MeOH	30	25	0.82	0.04
TsOYE	MeOH	35	25	0.75	0.05
TsOYE	MeOH	40	25	0.58	0.03
TsOYE	MeOH	45	25	0.47	0.02
TsOYE	MeOH	no enzyme	25	0.05	0.01
TsOYE	MeOH	no substrate	25	0.13	0.02
TsOYE	MeOH	0	30	1.47	0.11
TsOYE	MeOH	5	30	1.39	0.02
TsOYE	MeOH	10	30	1.33	0.07
TsOYE	MeOH	15	30	1.34	0.11
TsOYE	MeOH	20	30	1.26	0.03
TsOYE	MeOH	25	30	1.12	0.06
TsOYE	MeOH	30	30	0.94	0.08
TsOYE	MeOH	35	30	0.79	0.03
TsOYE	MeOH	40	30	0.62	0.01
TsOYE	MeOH	45	30	0.41	0.03
TsOYE	MeOH	no enzyme	30	0.05	0.01
TsOYE	MeOH	no substrate	30	0.18	0.06
TsOYE	MeOH	0	35	1.48	0.05
TsOYE	MeOH	5	35	1.53	0.01
TsOYE	MeOH	10	35	1.44	0.09
TsOYE	MeOH	15	35	1.40	0.06
TsOYE	MeOH	20	35	1.37	0.07
TsOYE	MeOH	25	35	1.22	0.02
TsOYE	MeOH	30	35	1.04	0.02
TsOYE	MeOH	35	35	0.89	0.06
TsOYE	MeOH	40	35	0.62	0.04
TsOYE	MeOH	45	35	0.48	0.02
TsOYE	MeOH	no enzyme	35	0.06	0.01
TsOYE	MeOH	no substrate	35	0.27	0.03
TsOYE	MeOH	0	40	1.49	0.10
TsOYE	MeOH	5	40	1.61	0.12
TsOYE	MeOH	10	40	1.53	0.08

TsOYE	MeOH	15	40	1.46	0.10
TsOYE	MeOH	20	40	1.36	0.03
TsOYE	MeOH	25	40	1.26	0.03
TsOYE	MeOH	30	40	1.09	0.06
TsOYE	MeOH	35	40	0.93	0.02
TsOYE	MeOH	40	40	0.63	0.03
TsOYE	MeOH	45	40	0.37	0.08
TsOYE	MeOH	no enzyme	40	0.11	0.03
TsOYE	MeOH	no substrate	40	0.27	0.03
TsOYE	MeOH	0	45	1.27	0.26
TsOYE	MeOH	5	45	1.59	0.13
TsOYE	MeOH	10	45	1.49	0.02
TsOYE	MeOH	15	45	1.52	0.04
TsOYE	MeOH	20	45	1.57	0.13
TsOYE	MeOH	25	45	1.36	0.07
TsOYE	MeOH	30	45	1.20	0.06
TsOYE	MeOH	35	45	0.95	0.03
TsOYE	MeOH	40	45	0.67	0.04
TsOYE	MeOH	45	45	0.32	0.00
TsOYE	MeOH	no enzyme	45	0.08	0.02
TsOYE	MeOH	no substrate	45	0.34	0.07
YqiG	EtOH	0	25	0.80	0.08
YqiG	EtOH	5	25	0.77	0.03
YqiG	EtOH	10	25	0.95	0.13
YqiG	EtOH	15	25	0.96	0.05
YqiG	EtOH	20	25	0.99	0.04
YqiG	EtOH	25	25	0.88	0.02
YqiG	EtOH	30	25	0.63	0.02
YqiG	EtOH	35	25	0.45	0.03
YqiG	EtOH	40	25	0.23	0.04
YqiG	EtOH	45	25	0.11	0.02
YqiG	EtOH	no enzyme	25	0.00	0.03
YqiG	EtOH	no substrate	25	0.00	0.11
YqiG	EtOH	0	30	0.56	0.02
YqiG	EtOH	5	30	0.66	0.07
YqiG	EtOH	10	30	0.67	0.02
YqiG	EtOH	15	30	0.85	0.11
YqiG	EtOH	20	30	0.75	0.02
YqiG	EtOH	25	30	0.63	0.08
YqiG	EtOH	30	30	0.44	0.03
YqiG	EtOH	35	30	0.25	0.05
YqiG	EtOH	40	30	0.10	0.01
YqiG	EtOH	45	30	0.06	0.01
YqiG	EtOH	no enzyme	30	0.00	0.03
YqiG	EtOH	no substrate	30	0.01	0.05
YqiG	EtOH	0	35	0.24	0.07
YqiG	EtOH	5	35	0.27	0.00

YqiG	EtOH	10	35	0.28	0.02
YqiG	EtOH	15	35	0.21	0.04
YqiG	EtOH	20	35	0.17	0.03
YqiG	EtOH	25	35	0.08	0.02
YqiG	EtOH	30	35	0.04	0.00
YqiG	EtOH	35	35	0.02	0.01
YqiG	EtOH	40	35	0.04	0.01
YqiG	EtOH	45	35	0.00	0.01
YqiG	EtOH	no enzyme	35	0.03	0.00
YqiG	EtOH	no substrate	35	0.01	0.02
YqiG	EtOH	0	40	0.15	0.13
YqiG	EtOH	5	40	0.05	0.02
YqiG	EtOH	10	40	0.04	0.05
YqiG	EtOH	15	40	0.03	0.01
YqiG	EtOH	20	40	0.03	0.01
YqiG	EtOH	25	40	0.03	0.02
YqiG	EtOH	30	40	0.04	0.02
YqiG	EtOH	35	40	0.04	0.01
YqiG	EtOH	40	40	0.02	0.05
YqiG	EtOH	45	40	0.04	0.03
YqiG	EtOH	no enzyme	40	0.00	0.00
YqiG	EtOH	no substrate	40	0.00	0.03
YqiG	MeOH	0	25	0.66	0.01
YqiG	MeOH	5	25	0.61	0.02
YqiG	MeOH	10	25	0.69	0.06
YqiG	MeOH	15	25	0.65	0.00
YqiG	MeOH	20	25	0.66	0.05
YqiG	MeOH	25	25	0.60	0.01
YqiG	MeOH	30	25	0.55	0.05
YqiG	MeOH	35	25	0.44	0.01
YqiG	MeOH	40	25	0.20	0.01
YqiG	MeOH	45	25	0.04	0.00
YqiG	MeOH	no enzyme	25	0.00	0.01
YqiG	MeOH	no substrate	25	0.00	0.07
YqiG	MeOH	0	30	0.51	0.07
YqiG	MeOH	5	30	0.42	0.05
YqiG	MeOH	10	30	0.41	0.03
YqiG	MeOH	15	30	0.43	0.06
YqiG	MeOH	20	30	0.40	0.03
YqiG	MeOH	25	30	0.40	0.02
YqiG	MeOH	30	30	0.28	0.06
YqiG	MeOH	35	30	0.21	0.01
YqiG	MeOH	40	30	0.11	0.03
YqiG	MeOH	45	30	0.04	0.01
YqiG	MeOH	no enzyme	30	0.00	0.03
YqiG	MeOH	no substrate	30	0.00	0.02
YqiG	MeOH	0	35	0.25	0.04

YqiG	MeOH	5	35	0.20	0.09
YqiG	MeOH	10	35	0.14	0.08
YqiG	MeOH	15	35	0.10	0.07
YqiG	MeOH	20	35	0.10	0.02
YqiG	MeOH	25	35	0.05	0.00
YqiG	MeOH	30	35	0.04	0.01
YqiG	MeOH	35	35	0.02	0.01
YqiG	MeOH	40	35	0.07	0.03
YqiG	MeOH	45	35	0.04	0.02
YqiG	MeOH	no enzyme	35	0.00	0.01
YqiG	MeOH	no substrate	35	0.00	0.06
YqiG	MeOH	0	40	0.12	0.12
YqiG	MeOH	5	40	0.09	0.03
YqiG	MeOH	10	40	0.04	0.01
YqiG	MeOH	15	40	0.02	0.01
YqiG	MeOH	20	40	0.03	0.01
YqiG	MeOH	25	40	0.02	0.01
YqiG	MeOH	30	40	0.03	0.02
YqiG	MeOH	35	40	0.02	0.02
YqiG	MeOH	40	40	0.03	0.01
YqiG	MeOH	45	40	0.05	0.03
YqiG	MeOH	no enzyme	40	0.01	0.02
YqiG	MeOH	no substrate	40	0.00	0.02

Supplementary References

1. Reich, S., Kress, N., Nestl, B. M. & Hauer, B. Variations in the stability of NCR ene reductase by rational enzyme loop modulation. *J. Struct. Biol.* **185**, 228–233 (2014).

2. Yanto, Y., Yu, H. H., Hall, M. & Bommarius, A. S. Characterization of xenobiotic reductase A (XenA): study of active site residues, substrate spectrum and stability. *Chem. Commun.* **46**, 8809–8811 (2009).

3. Schittmayer, M. *et al.* Old Yellow Enzyme-Catalyzed Dehydrogenation of Saturated Ketones. *Adv. Synth. Catal.* **353**, 268–274 (2011).
